# Tumor-intrinsic IRE1α signaling controls protective immunity in lung cancer

**DOI:** 10.1038/s41467-022-35584-9

**Published:** 2023-01-09

**Authors:** Michael J. P. Crowley, Bhavneet Bhinder, Geoffrey J. Markowitz, Mitchell Martin, Akanksha Verma, Tito A. Sandoval, Chang-Suk Chae, Shira Yomtoubian, Yang Hu, Sahil Chopra, Diamile A. Tavarez, Paolo Giovanelli, Dingcheng Gao, Timothy E. McGraw, Nasser K. Altorki, Olivier Elemento, Juan R. Cubillos-Ruiz, Vivek Mittal

**Affiliations:** 1 Department of Cardiothoracic Surgery, Weill Cornell Medicine, 525 East 68th street, New York, NY 10065 USA; 2 Neuberger Berman Lung Cancer Center, Weill Cornell Medicine, 525 East 68th street, New York, NY 10065 USA; 3 Weill Cornell Graduate School of Medical Sciences, Weill Cornell Medicine, 525 East 68th street, New York, NY 10065 USA; 4 Caryl and Israel Englander Institute for Precision Medicine, Weill Cornell Medicine, 413 East 69th street, New York, NY 10065 USA; 5 HRH Prince Alwaleed Bin Talal Bin Abdulaziz Alsaud Institute for Computational Biomedicine, Department of Physiology and Biophysics, Weill Cornell Medicine, 525 East 68th street, New York, NY 10065 USA; 6 Department of Cell and Developmental Biology, Weill Cornell Medicine, 525 East 68th street, New York, NYk 10065 USA; 7 Sandra and Edward Meyer Cancer Center, Weill Cornell Medicine, 413 East 69th street, New York, NY 10065 USA; 8 Department of Obstetrics and Gynecology, Weill Cornell Medicine, 525 East 68th street, New York, NY 10065 USA; 9 Immunology and Microbial Pathogenesis Program, Weill Cornell Medicine, 525 East 68th street, New York, NY 10065 USA; 10 Department of Biochemistry, Weill Cornell Medicine, 525 East 68th street, New York, NY 10065 USA; 11 Present Address: Volastra Therapeutics, New York, NY 10027 USA; 12 Present Address: Salk Institute for Biological Studies, San Diego, CA USA; 13 Present Address: Vertex Ventures HC, 345 California Avenue, Palo Alto, CA 94306 USA; 14 Present Address: Regeneron Pharmaceuticals, 777 Old Saw Mill River Rd, Tarrytown, NY 10591 USA

**Keywords:** Gene regulation, Lung cancer, Non-small-cell lung cancer

## Abstract

IRE1α-XBP1 signaling is emerging as a central orchestrator of malignant progression and immunosuppression in various cancer types. Employing a computational *XBP1s* detection method applied to TCGA datasets, we demonstrate that expression of the *XBP1s* mRNA isoform predicts poor survival in non-small cell lung cancer (NSCLC) patients. Ablation of IRE1α in malignant cells delays tumor progression and extends survival in mouse models of NSCLC. This protective effect is accompanied by alterations in intratumoral immune cell subsets eliciting durable adaptive anti-cancer immunity. Mechanistically, cancer cell-intrinsic IRE1α activation sustains mPGES-1 expression, enabling production of the immunosuppressive lipid mediator prostaglandin E_2_. Accordingly, restoring mPGES-1 expression in IRE1α^KO^ cancer cells rescues normal tumor progression. We have developed an IRE1α gene signature that predicts immune cell infiltration and overall survival in human NSCLC. Our study unveils an immunoregulatory role for cancer cell-intrinsic IRE1α activation and suggests that targeting this pathway may help enhance anti-tumor immunity in NSCLC.

## Abstract (web summary)

The IRE1α-XBP1 arm of the unfolded protein response (UPR) has been associated with immunosuppression and cancer progression. Here the authors show that IRE1α-XBP1 activation is associated with poor overall survival in patients with non-small cell lung cancer and that IRE1α loss in cancer cells promotes anti-tumor immune responses in lung cancer preclinical models.

## Introduction

Despite advancements in surgeries and the availability of FDA-approved molecular targeted therapies, the mortality in NSCLC remains high^1,2^. Recently, immunotherapies, particularly those targeting the PD-1/PDL-1 axis, have offered significant improvements in overall survival for a subset of patients with NSCLC^2^. However, a majority (>75%) of patients experience little clinical benefit^3^ due to a variety of immunosuppressive barriers in the TME^2^. Given the unmet medical need, a major focus is to identify and characterize additional immunosuppressive mechanisms^4^.

A pathway that has recently been appreciated for its immunomodulatory capacity is the unfolded protein response (UPR)^5,6^. Adverse conditions in the TME such as hypoxia, nutrient starvation, and oxidative stress disrupt the protein folding capacity of the endoplasmic reticulum (ER) in infiltrating cells, provoking a state of “ER stress” that activates the UPR to restore proteostasis in this organelle^5,7^. Activated during periods of ER stress, the inositol-requiring enzyme 1 (IRE1α) endoribonuclease domain excises a 26-nucleotide fragment from the primary *XBP1* mRNA, generating the spliced isoform *XBP1s* that encodes the functionally active transcription factor XBP1s, inducing UPR target gene expression^8^. XBP1s in breast cancer cells has been shown to drive malignant progression by promoting tumor cell survival and metastasis under hypoxic conditions^9,10^, and previously, we reported that aberrant IRE1α-XBP1 signaling in intratumoral leukocytes facilitated immune escape and metastasis in ovarian cancer^11,12^. Nonetheless, it remains elusive whether direct IRE1α activation in malignant cells controls the tumor immune microenvironment and adaptive antitumor immunity. The UPR has previously been investigated in the context of chronic obstructive pulmonary disease and cystic fibrosis^13^, and a recent study showed that XBP1s-mediated upregulation of insulin-like growth factor binding protein-3 (IGFBP3) promotes NSCLC invasion and metastasis^14^. However, the immunomodulatory role of IRE1α-XBP1 signaling in NSCLC remains largely unexplored.

Here, we show *XBP1s* expression is associated with poor outcome in NSCLC patients. Using KRAS mouse models of NSCLC, we uncover that cancer cell-intrinsic IRE1α fosters marked intratumoral immunosuppression that facilitates malignant progression.

## Results

### IRE1α-XBP1 activation is associated with poor overall survival in human NSCLC

**Fig. 1:**
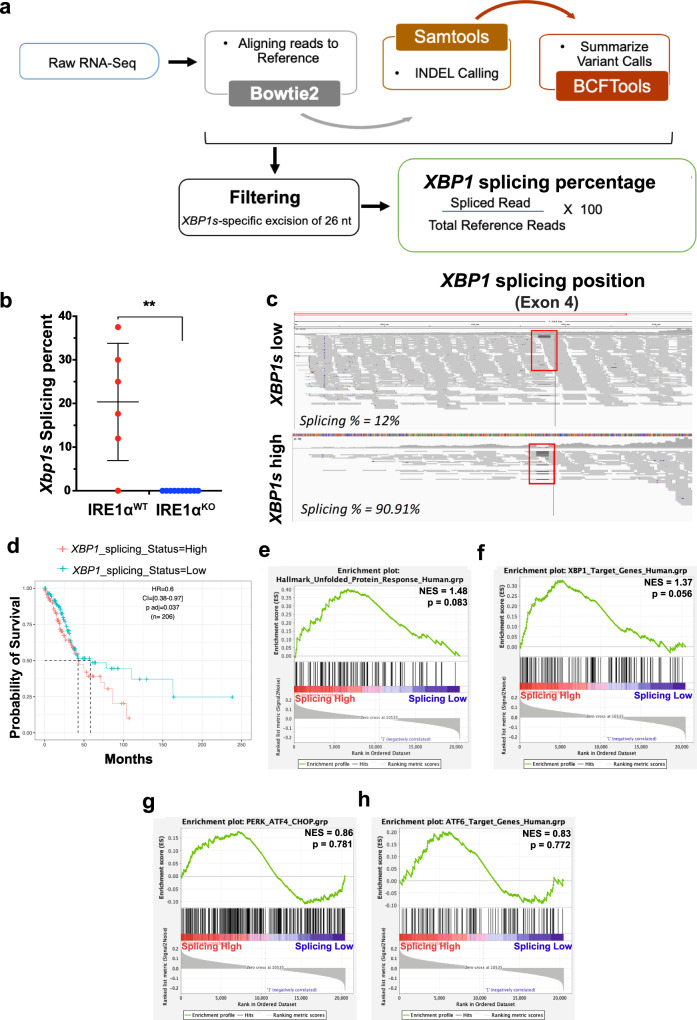
*XBP1s* is associated with decreased overall survival in human NSCLC. **a** Schematic of the RNA-seq based *XBP1s* detection pipeline. **b** Box and whisker plot of computationally evaluated *Xbp1* splicing in isogenic *IRE1α*^WT^ (red, *n* = 6) or IRE1α^KO^ (blue, *n* = 10) HKP1 cancer cells. Data were presented as mean ± SD. *P* < 0.0002. Unpaired, two-tailed, Student’s *t*-test. **c** Visual survey of aligned reads using Integrative Genomic Viewer (IGV) showing detected indels including the 25 nucleotide excision of the *XBP1* gene (red box). Representative *XBP1s* low and *XBP1s* high samples are shown. **d** Kaplan–Meier survival plots depicting associations between overall survival (OS) and *XBP1s* status in human TCGA-LUAD. High is top 1/3rd (*n* = 103) and low is bottom 1/3rd (103) of the *XBP1*splicing scores. The high *XBP1s* group is the reference population. HR is the hazard ratio and *p* adj is the log-rank *p* value from the multivariate Cox proportional-hazards regression model adjusted for age at diagnosis, gender, pathology, TN stages, and smoking history. **e**–**h** GSEA hyperparametric curves showing expression of genes controlled by the UPR (**e**), XBP1s (**f**), PERK (**g**), and ATF6 (**h**) in patients with high vs. low *XBP1s* levels. Source data are provided as a Source Data file.

To determine the clinical relevance of IRE1α-XBP1 signaling in NSCLC patients, we developed a computational pipeline to specifically quantify the percentage of the spliced *XBP1* mRNA isoform (*XBP1s*) relative to the total *XBP1* transcript from RNA-seq data available in the TCGA database (Fig. 1a). Briefly, short reads were aligned to the *XBP1* unspliced transcript using Bowtie2 parameters that depenalize long gaps, followed by estimation of the fraction of spliced reads over read coverage around the *XBP1* splicing event (Supplementary Fig. 1a). We demonstrated the sensitivity of this strategy using RNA-seq data from isogenic IRE1α^WT^ and IRE1α^KO^ cell lines (Fig. 1b). *XBP1s* abundance in the TCGA dataset of NSCLC LUAD patients (*n* = 232) was evaluated (Fig. 1c), and the top and bottom tertiles were compared. Patients with low *XBP1s* were associated with significantly improved overall survival (OS) compared to high *XBP1s* (Fig. 1d, HR = 0.6, *p* < 0.037), *XBP1s* high median 37.6 months, *XBP1s* low median 58.3 months). Clinicopathologic parameters (gender, smoking status, pathologic stage, T stage, N stage, and neoadjuvant therapy) did not correlate with *XBP1s* (Supplementary Table 1), and similar trends were observed in a multivariate analysis using Cox proportional-hazards regression model adjusted for age at the time of diagnosis, gender, pathology, and TN stages (Supplementary Table 2). To demonstrate that the difference in survival was specific to the IRE1α-XBP1 axis but not due to a broader UPR response, we performed Gene Set Enrichment Analysis (GSEA), comparing the top and bottom *XBP1s* tertiles against UPR signatures derived from the Molecular Signatures Database (MSigDB), and Harmonizome. Both the Hallmark UPR signature and *XBP1s* target genes were enriched in the *XBP1s* high patient cohort (Fig. 1e, f), whereas genes controlled by other sensors of ER stress including protein kinase RNA (PKR)-like ER Kinase (PERK) and activating transcription factor 6 (ATF6)^15^ were not significantly enriched (Fig. 1g, h). Additionally, we did not observe differential expression of Regulated IRE1α-Dependent Decay (RIDD) targets between these groups (Supplementary Fig. 1b). These findings demonstrate *XBP1s* level as prognostic of reduced survival in human NSCLC.

### IRE1α loss in cancer cells delays tumor growth and improves survival in mouse models of NSCLC

**Fig. 2:**
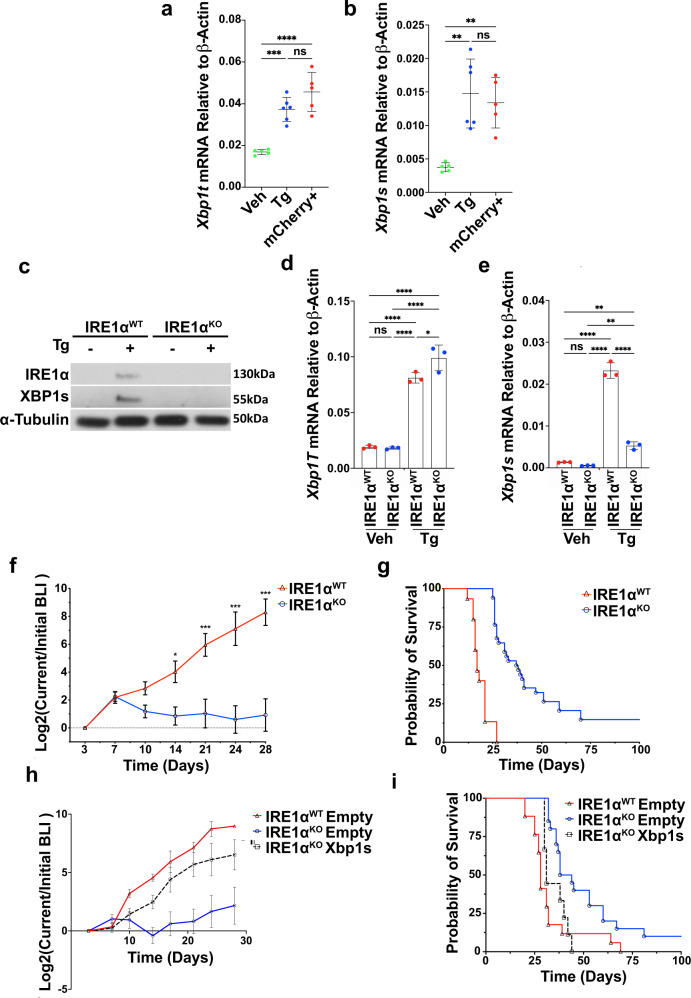
IRE1α deficiency in the cancer cell impairs HKP1 tumor growth and extends host survival. **a**, **b** RT-PCR of total *Xbp1* (**b**, Veh vs Tg, *P* = 0.0004; Veh vs mCherry, *P* < 0.0001; Tg vs mCherry, *P* = 0.111) and *Xbp1s* (**c** Veh vs Tg, *P* = 0.001; Veh vs mCherry, *P* = 0.0042, Tg vs mCherry, *P* = 0.8234), in vehicle (*n* = 5), or 1 µM Tg for 6 h (*n* = 3) and mCherry+ HKP1 cells from tumors (*n* = 5). Data were presented as mean ± SD. Tg Thapsigargin. One-way ANOVA with Tukey’s multiple comparisons test for ratios; *P < 0.05, **P < 0.001, and ***P < 0.0001. **c** Western blot of IRE1α and XBP1s in IRE1α^WT^ or IRE1α^KO^ HKP1 cells treated with vehicle or 1 μM Tg for 6 h. This result represents three replicates. **d**, **e** RT-PCR for of *Xbp1* total (**e** Veh WT vs Tg WT, *P* < 0.0001; Veh WT vs KO Veh, *P* = 0.9970; Veh WT vs KO Tg, *P* < 0.0001; Tg WT vs KO Veh, *P* < 0.0001; Tg WT vs KO Tg, *P* = 0.0343; KO Veh vs KO Tg, *P* < 0.0001), *Xbp1*s (**f** Veh WT vs Tg WT, *P* < 0.0001; Veh WT vs KO Veh, *P* = 0.7959; Veh WT vs KO Tg, *P* = 0.0071; Tg WT vs KO Veh, *P* < 0.0001; Tg Wt vs KO Tg, *P* < 0.0001; KO Veh vs KO Tg, *P* = 0.0023) in *IRE1α*^WT^ or *IRE1α*^KO^ HKP1 cells treated with vehicle (*n* = 3) or 1 μM Tg (*n* = 3) for 6 h. One-way ANOVAs with Tukey’s multiple comparisons test; *P < 0.05, **P < 0.001, and ***P < 0.0001. Data were shown as mean ± SD. **f** BLI plots of longitudinally tracked in vivo *IRE1α*^WT^ (red, *n* = 6) vs *IRE1α*^KO^ (blue, *n* = 10) HKP1 tumors (Day 3, *P* = NS; Day 7, *P* = NS; Day 10, *P* = NS; Day 14, *P* = 0.0275; Day 21, *P* = 0.0002; Day 24, *P* < 0.0001 and Day 28, *P* < 0.0001). Data were shown as mean ± SEM of biological replicates. Analyses of different time points in tumor progression were performed using two-way ANOVA with Tukey’s multiple comparisons test; *P < 0.05, **P < 0.001, ***P < 0.0001. **g** Kaplan–Meier plots showing the probability of overall survival in *IRE1α*^WT^ (red, *n* = 15) vs *IRE1α*^KO^ (blue, *n* = 35) HKP1 tumor-bearing mice *(P* < 0.001*)*. Tumors were allowed to progress until endpoint and survival were evaluated using Mantel–Haenszel Log-rank-test). **h** BLI plots of longitudinally tracked in vivo *IRE1α*^WT^ empty vector (red, *n* = 5), *IRE1α*^KO^ empty vector (blue, *n* = 5) and *IRE1α*^KO^ expressing *Xbp1s* cDNA (black, *n* = 5) HKP1 tumors. Data were shown as mean ± SEM of biological replicates. Data were pooled from two independent experiments. *(P* < 0.001 for *IRE1α*^*WT*^ vs. *IRE1α*^KO^*; P* < 0.002 for *IRE1α*^KO^ vs. *IRE1α*^KO^ expressing *Xbp1s* cDNA at day 28). Data were shown as mean ± SEM of biological replicates. Analyses of different time points in tumor progression were performed using two-way ANOVA with Tukey’s multiple comparisons test. **i** Kaplan–Meier plots showing overall survival in *IRE1α*^WT^ (red, *n* = 17), *IRE1α*^KO^ (blue *n* = 20), and *IRE1α*^KO^ expressing *Xbp1s* cDNA (black, *n* = 9). Tumors were allowed to progress until endpoint and survival were evaluated using Mantel–Haenszel Log-rank-test). Data were pooled from two independent experiments. (*P* = 0.0009 for *IRE1α*^WT^ vs. *IRE1α*^KO^; and *P* = 0.0058 for *IRE1α*^KO^ vs*. IRE1α*^KO^ *Xbp1s*. Source data are provided as a Source Data file.

We next investigated the functional role of IRE1α-XBP1 signaling in NSCLC. We employed the mutant *Kras*^*G12D*^*p53*^−*/*−^ (HKP1) orthotopic model of lung cancer, which develops adenocarcinoma with histopathological similarities to human NSCLC, in syngeneic immunocompetent C57BL/6 mice^16^. Analysis of differentially upregulated genes between HKP1 tumor epithelial cells and normal adjacent lung epithelial cells sorted from in vivo^16^ revealed enrichment of UPR-related categories in HKP1 cells (Supplementary Fig. 2a), associated with enrichment of UPR transcription factors (Supplementary Fig. 2b). Having demonstrated increased UPR signaling in HKP1 tumor cells compared to normal lung resident epithelial cells in vivo, we analyzed the status of IRE1α-XBP1 signaling. As expected, relative to vehicle controls, there was strong activation of IRE1α-XBP1 in mCherry+ tumor cells sorted from HKP1 tumors, comparable to HKP1 cells experiencing thapsigargin (Tg)-induced ER stress in vitro, (Fig. 2a, b). Activation of IRE1α-XBP1 was associated with a marked upregulation of XBP1s target genes including *Erdj4, Sec61a1, Sec24d, Edem1*, and *Hyou1* (Supplementary Fig. 2c–g). HKP1 tumor cell sorted from mouse lungs did not show activation of ATF6 or PERK branches of the UPR compared to Tg-treated cells (Supplementary Fig. 2h–k).

Given the selective activation of IRE1α-XBP1 lung cancer cells, we used CRISPR-Cas9 to determine the impact of IRE1α loss on HKP1 tumor progression. To avoid the stable expression of immunogenic Cas9, we electroporated fluorescently labeled CRISPR/Cas9 ribonucleoproteins targeting the gene encoding IRE1α in HKP1 tumor cells using the NEON system (Thermo Fisher)^17,18^. Single-cell colonies (Supplementary Fig. 3a) were expanded and evaluated for IRE1α^KO^ and IRE1α^WT^ (non-targeting sgRNA control) by Sanger sequencing, flow cytometry with XBP1s-specific antibody, and qPCR (Supplementary Fig. 3b–e). As expected, expression of the IRE1-XBP1s target gene *Erdj4* was reduced in IRE1α^KO^, whereas IRE1α -independent UPR markers BiP and CHOP remained unchanged (Supplementary Fig. 3f–h). Ten independent IRE1α^KO^ single-cell colonies were pooled and confirmed for XBP1s deficiency following Tg treatment compared to IRE1α^WT^ cells. The total *Xbp1* isoform was increased in both IRE1α^KO^ and IRE1α^WT^ cells. However, as expected upon induction of ER stress, there was a significant reduction in *Xbp1s* in IRE1α^KO^ cells compared to controls as determined by western blot and RT-PCR (Fig. 2c–e). We confirmed that IRE1α^KO^ cells did not significantly upregulate XBP1s canonical downstream target genes following treatment with Tg (Supplementary Fig. 4a–d), nor did they induce compensatory activation of the global UPR programs (Supplementary Fig. 4e–h), or RIDD target genes (Supplementary Fig. 4i, j). IRE1α^KO^ did not impact proliferation, viability, apoptosis, invasion, or cell cycle in the absence of exogenous ER stress (Supplementary Fig. 5a–g). These data indicate that ablating IRE1α preserves the function of the other ER stress sensors to avoid proteotoxic stress in this organelle.

We next evaluated the impact of cancer cell-intrinsic IRE1α loss on HKP1 tumor progression. IRE1α^KO^ tumors progressed comparably to their IRE1α^WT^ counterparts until day 10. However, after this time, IRE1α deficiency in the cancer cell-induced tumor regression compared with IRE1α^WT^ controls (Fig. 2f and Supplementary Fig. 6a, b). Consistent with delayed tumor progression, IRE1α^KO^ tumor-bearing mice demonstrated a significant increase in overall survival compared to controls (Fig. 2g). To confirm these phenotypes in another model, we ablated IRE1α in the CMT-167 immunocompetent mouse NSCLC model^19^. Mice bearing CMT-167 IRE1α^KO^ tumor also showed a significant survival benefit compared with their IRE1α^WT^ counterparts (Supplementary Fig. 6c).

To determine whether the IRE1α^KO^ tumor phenotype was a result of XBP1s deficiency, we ectopically restored *Xbp1s* expression in IRE1α^KO^ HKP1 cells, and observed a rescue of tumor growth kinetics (Fig. 2h and Supplementary Fig. 6d). Furthermore, XBP1s-induced tumor growth in IRE1α^KO^ mice was associated with decreased survival, with median survival in IRE1α^WT^ (28 days), IRE1α^KO^ (41 days) and IRE1α^KO^ *Xbp1s* (31 days) (Fig. 2i). Together, these results reveal a major protumoral role of IRE1α-XBP1 signaling in NSCLC.

### Tumor-intrinsic IRE1α loss activates immune-related transcriptional programs

To define the mechanisms underlying the tumor regression phenotype caused by IRE1α loss, we performed RNA-seq of mCherry+ malignant epithelial cells sorted from IRE1α^WT^ or IRE1α^KO^ tumors at two different time points of growth: day 10, when tumor growth was comparable, and day 14, when tumors showed regression induced by IRE1α loss. Principal component analysis (PCA) of the RNA-seq data showed that 99% of the variance was captured within the first two components, and a robust segregation between the IRE1α^WT^ and IRE1α^KO^ tumors was observed at both time points (Supplementary Fig. 7a). As expected, RNA-seq data confirmed decreased expression of IRE1α-XBP1 canonical downstream targets in the absence of IRE1α (Supplementary Fig. 7b), while no significant impact on IRE1-independent UPR programs or RIDD targets (Supplementary Fig. 7c) were observed.

**Fig. 3:**
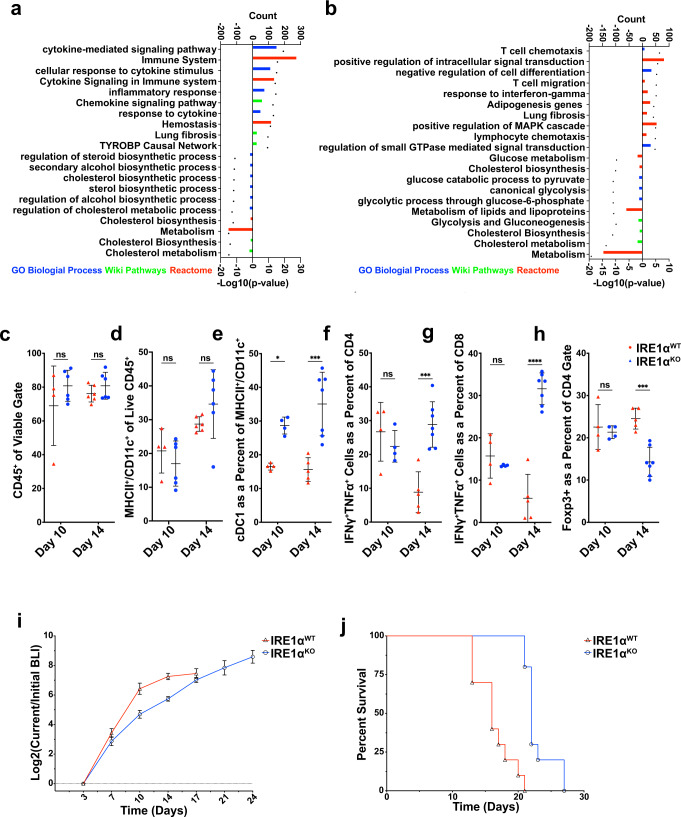
Adaptive immunity mediates the protective effects of tumor cell-intrinsic IRE1α loss. **a**, **b** Top ten upregulated and ten downregulated terms enriched from GO Biological Process (Blue), Wiki Pathways (Green) and Reactome (Red) in differentially expressed genes between *IRE1α*^*WT*^ vs *IRE1α*^*KO*^ mcherry+ cells from HKP1 tumors at day 10 (**a**) and 14 (**b**). Significance cutoff values were set at log2 fold-change >0.5, *p* value < 0.05 and false discovery rate <10%). The count of genes enriching the GO term is represented as a barplot, and the plotted –log10 *p* values are represented as a dot above its corresponding bar. Genes matching these criteria were analyzed using the Enrichr portal with standard parameters. **c**–**h** Box and whisker plots of flow cytometry data from *IRE1α*^WT^ (red) vs *IRE1α*^KO^ (blue) HKP1 tumor-bearing lungs at day 10 (*n* = 4 *IRE1α*^WT^ and *n* = 6 *IRE1α*^KO^) and 14 (*n* = 6 *IRE1α*^WT^ and *n* = 7 *IRE1α*^KO^), showing percent viable CD45+ (**c**), MHCII^+^ CD11C^+^ (**d**), and cDC1 (**e**). IFNγ/TNFα^+^ T cells as a percent of CD4 (**f**), IFNγ/TNFα^+^ T cells as a percent of CD8 (**g**), and percent of T-regulatory cells of viable CD45+ cells (**h**). Data were shown as mean ± SD. Two-way ANOVA with Tukey’s multiple comparisons test; *P < 0.05, **P < 0.001, and ***P < 0.000, ns non-significant. **i** *IRE1α*^WT^ or *IRE1α*^KO^ HKP1 tumor growth in Rag2-KO mice. Data were shown as mean ± SEM of biological replicates, (*n* = 5, per condition). Analyses of different time points in tumor progression were performed using two-way ANOVA with Tukey’s multiple comparisons test; *P < 0.05, **P < 0.001, and ***P < 0.0001; ns non-significant. **j** Kaplan–Meier plots showing probability of overall survival in Rag2-KO mice bearing *IRE1α*^*WT*^ or *IRE1α*^*KO*^ HKP1 tumors. *(P* < 0.001, *n* = 5 per condition). Source data are provided as a Source Data file.

Comparison of IRE1α^WT^ and IRE1α^KO^ HKP1 transcriptomes at days 10 and 14 identified 2738 and 2712 differentially regulated genes, respectively (Supplementary Fig. 7d, e and Supplementary Data 1). To explore the mechanisms driving the tumor regression phenotype, we employed Enrichr^20^, which queries multiple pathway and ontology databases. Unexpectedly, analysis of differentially regulated genes (days 10 and 14) revealed enrichment of immune-related categories in the upregulated genes from IRE1α^KO^ samples (cytokine signaling/activity, T cell chemotaxis, migration, response to interferon-gamma, immune system activation, inflammatory responses, and neutrophil activation) (Fig. 3a, b and Supplementary Data 2). In contrast, IRE1α^WT^ cancer cells demonstrated enrichment in cell cycle, metabolism, and cholesterol and steroid biosynthesis gene programs (Fig. 3a, b and Supplementary Data 2). The enrichment of immune pathways in the IRE1α^KO^ arm suggested that cancer cell-intrinsic loss of IRE1α may have reprogramed the immune microenvironment of HKP1 tumors.

### Adaptive antitumor immunity is enhanced in IRE1α^KO^ tumors

Loss of IRE1α in HKP1 tumors did not affect the total number of infiltrating CD45+ cells (Fig. 3c and Supplementary Fig. 8), but it drastically altered the proportion of diverse intratumoral immune cell subsets with a significant increase in type 1 conventional DCs (cDC1:CD11b^−^, CD11c^+^, MHCII^+^, CD64^Low^ CD24^High^ CD103^+^) and type 2 conventional DCs (cDC2:(CD11c^+^, MHCII^+^, CD11b^+^, CD103^−^, CD64^Low^ CD24^High^) (Fig. 3d, e and Supplementary Fig. 8d, h, i). IRE1α^KO^ tumors also showed a decrease in neutrophils/polymorphonuclear myeloid cells (PMN-MCs; CD11b^+^ CD11c^−^ Ly6c^−^ Ly6g^+^) (Supplementary Fig. 8e, h, i), with no significant change in monocytic MDSC (Mo-MDSC; CD11b^+^ CD11c^−^ Ly6c^+^ Ly6g^−^) (Supplementary Fig. 8f–i), or CD11b^+^ F4/80^**+**^ macrophages (Supplementary Fig. 8g–i). Regarding lymphoid populations, IRE1α^WT^ and IRE1α^KO^ tumors showed comparable numbers of CD3, CD4 and CD8 T cells (Supplementary Figs. 9a–d,  8b, c). However, IRE1α-deficient tumors exhibited a significant increase in the proportion of intratumoral CD4 + and CD8 + T cells producing the effector cytokines TNFα and IFNγ in vivo (Fig. 3f, g), with a concomitant decrease in immunosuppressive Foxp3+ Tregs (Fig. 3h and Supplementary Fig. 8b, c). These intratumoral CD4 and CD8 T cells also demonstrated increased production of TNFα and IFNγ on a per-cell basis (Supplementary 9e–j). Hence, the immunophenotyping data suggest the activation of an adaptive antitumor immune response caused by IRE1α loss, which manifests at day 14 in concordance with the observed tumor regression phenotype.

To functionally determine if the tumor regression phenotype was mediated by adaptive antitumor immunity, we implanted IRE1α^WT^ or IRE1α^KO^ cancer cells into wildtype or Rag2-deficient hosts lacking T and B cells. Strikingly, Rag2 knockout mice lacking an adaptive immune system failed to show tumor regression and demonstrated a marked reduction in overall survival upon challenge with IRE1α^KO^ HKP1 tumors (Fig. 3i, j), compared with their immune-competent counterparts (Fig. 2f, g). Further, to confirm that implantation of IRE1α^KO^ HKP1 cancer cells elicited durable antitumor immunity, we performed tumor rechallenge experiments. Indeed, long-term survivors initially harboring IRE1α^KO^ tumors in Fig. 1i were protected upon rechallenge with WT HKP1 cells, compared with age-matched tumor naïve controls (Supplementary Fig. 10).

Together, these data indicate that cancer cell-intrinsic IRE1α-XBP1s may curtail the tumoricidal capabilities of adaptive immune cells in the TME, hence promoting immune evasion and malignant progression. Furthermore, activation of adaptive antitumor immune responses in IRE1α^KO^ tumors is consistent with emerging evidence in melanoma and glioblastoma indicating that cancer cell-intrinsic pathways alter immune cells in the TME^21^.

### IRE1α loss controls the TME by sustaining local PGE_2_ production

**Fig. 4:**
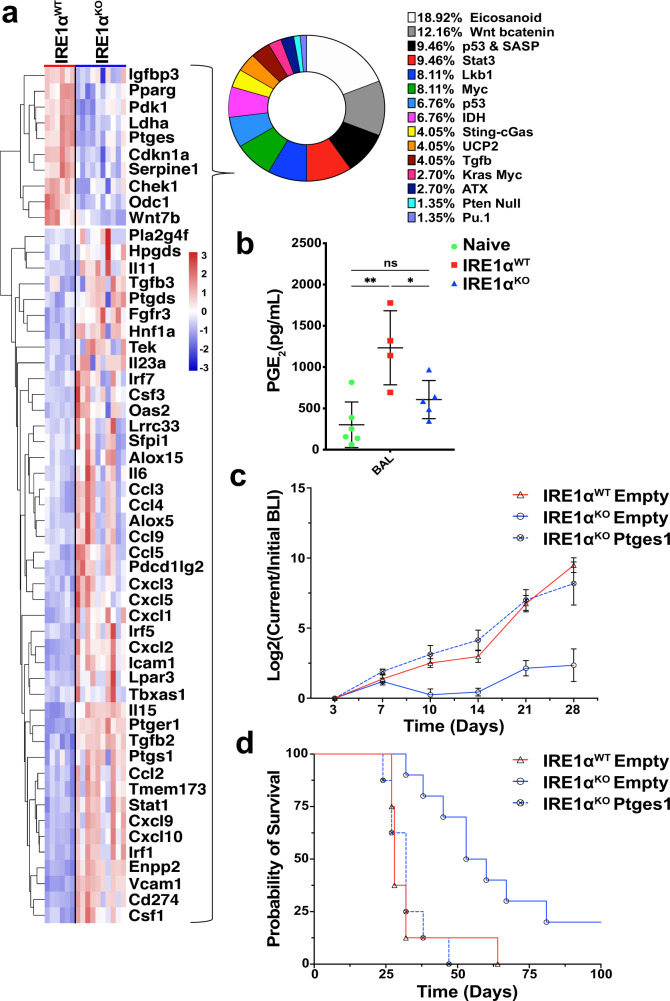
IRE1α-XBP1 signaling drives immunosuppressive PGE_2_ production that promotes NSCLC progression. **a** Heatmap of differentially expressed genes between *IRE1α*^KO^ vs. *IRE1α*^WT^ HKP1 cells from the Immunomodulator database. **b** BAL PGE_2_ levels as measured by ELISA from tumor naïve (green, *n* = 6), and *IRE1α*^WT^ (red, *n* = 4), or *IRE1α*^KO^ (blue, *n* = 5) tumor-bearing lungs. Data were shown as mean ± SD. One-way ANOVA with Tukey’s multiple comparisons test for ratios; *P < 0.05, **P < 0.001, and ***P < 0.0001. **c** BLI plots of longitudinally tracked in vivo *IRE1α*^WT^ empty vector (red, *n* = 10), *IRE1α*^KO^ empty vector (blue, *n* = 10) and *IRE1α*^KO^ *Ptges* cDNA (blue broken line, *n* = 10) HKP1 tumors. Data were shown as mean ± SEM of biological replicates. Analyses of different time points in tumor progression were performed using two-way ANOVA with Tukey’s multiple comparisons test; *P < 0.05, **P < 0.001, and ***P < 0.0001. **d** Kaplan–Meier plots showing the probability of overall survival in *IRE1α*^WT^ (red) vs *IRE1α*^KO^ (blue) and *IRE1α*^KO^ *Ptges* cDNA HKP1 tumor-bearing mice *(P* < 0.001*)*. Tumors were allowed to progress until the endpoint and survival were evaluated using Mantel–Haenszel Log-rank-test). Source data are provided as a Source Data file.

To understand how cancer cell-intrinsic IRE1α activation shapes the tumor immune microenvironment, we manually curated an Immunomodulator dataset comprised of known tumor-intrinsic signaling immunomodulatory pathways from published studies and reviews^21–23^ (Supplementary Data 3). Analysis of this database with differentially regulated genes identified from comparison of IRE1α^WT^ and IRE1α^KO^ HKP1 cells revealed that eicosanoid and WNT/β-catenin were the most enriched pathways in IRE1α^WT^ tumors (Fig. 4a and Supplementary Fig. 11). Consistently, eicosanoid biosynthesis was also identified in the unsupervised (Supplementary Data 2). Evaluation of the candidate genes in the eicosanoid biosynthetic pathway identified *Ptges* (encoding m-PGES1, prostaglandin E synthase), as one of the most significantly downregulated genes in IRE1α^KO^ cancer cells. m-PGES1 is an inducible enzyme that rapidly converts prostaglandin H2 (PGH_2_) to prostaglandin E2 (PGE_2_), a potent lipid mediator that is known to promote differentiation of Tregs^24^, enhance MDSC function^25,26^, and block DC differentiation, infiltration and activation^27^. Consistent with our RNA-seq findings, analysis of bronchoalveolar lavage fluid (BALF) showed that mice bearing IRE1α^KO^ HKP1 tumors had a marked decrease in PGE_2_ levels, compared with their IRE1α^WT^ tumor-bearing counterparts (Fig. 4b). *Ptges* transcript and secreted PGE_2_ levels were reduced in Tg-treated IRE1α^KO^ HKP1 cells compared with IRE1α^WT^ cells, confirming a direct link between IRE1α and PGE_2_ biosynthesis (Supplementary Fig. 12a, b). This direct link is reinforced by our recent demonstration that XBP1s can directly transactivate *COX2* and *PTGES* genes in human leukocytes to enable PGE_2_ production in the context of inflammatory pain^28^. Hence, we posited that PGE_2_ secreted by the tumor cells via IRE1-XBP1 activation may modulate the tumor immune microenvironment.

To directly establish the role of the IRE1α-PGE_2_ axis in malignant progression, we stably reconstituted *Ptges* in IRE1α^KO^ HKP1 cells (Supplementary Fig. 13a). As expected, *Ptges* expression did not alter total *Xbp1* or *Xbp1s* expression (Supplementary Fig. 13b, c), canonical IRE1α target genes (Supplementary Fig. 13d, e), or other UPR markers (Supplementary Fig. 13f, g). Ectopic *Ptges* expression did not impact HKP1 cancer cell viability either (Supplementary Fig. 13h). Hence, we next determined the effects of restoring *Ptges* expression in IRE1α^KO^ HKP1 cells in vivo. Normal tumor growth kinetics was observed in mice with IRE1α^KO^ tumors reconstituted with *Ptges* (Fig. 4c, *p* = 0.002, and Supplementary Fig. 13i). Furthermore, *Ptges*-induced tumor growth in IRE1α^KO^ mice was associated with decreased survival with a median survival of 28 days in IRE1α^WT^, 56.5 days in IRE1α^KO^ and 32 days in IRE1α^KO^ *Ptges* (32 days) (Fig. 4d).

Compared to IRE1α^KO^ tumors, IRE1α^KO^ tumors reconstituted with *Ptges* showed a trend towards an increase in Foxp3 + Tregs. There was also a trend towards a decrease in cytokine-producing CD4 and CD8 T cells (Supplementary Fig. 13j). Together, these results establish the role of the IRE1α-mPEGS1-PGE_2_ axis in experimental NSCLC progression and host survival.

### Mouse IRE1α signature predicts outcomes in human NSCLC

**Fig. 5:**
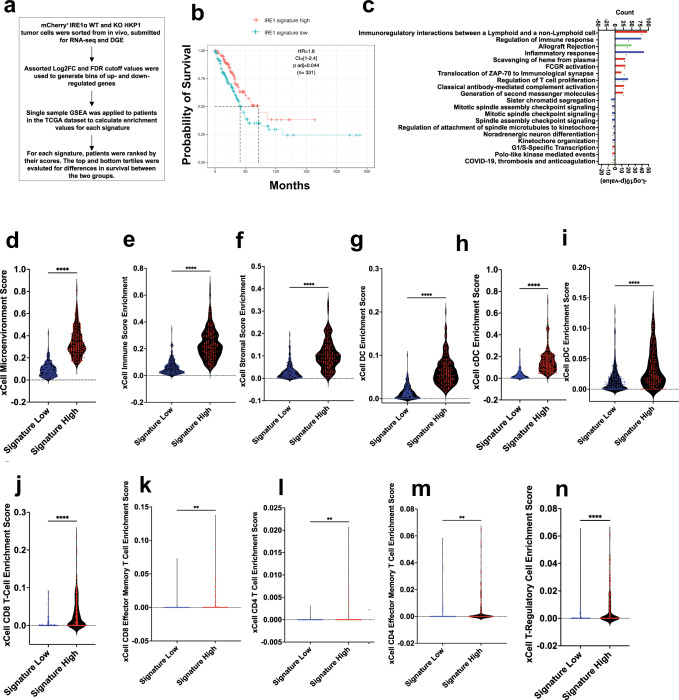
IRE1α^KO^ gene signature enrichment is associated with human LUAD survival and immune infiltration. **a** Schematic depicting IRE1α signature generation and evaluation. **b** Kaplan–Meier survival plots depicting associations between overall survival (OS) and IRE1α^KO^ signature status in human TCGA-LUAD. High is top 1/3rd (*n* = 166) and low is bottom 1/3^rd^ (*n* = 165) of the *IRE1α*^KO^ signature scores. HR is the hazard ratio and *p* adj is log-rank *p* value from the multivariate Cox proportional-hazards regression model. **c** Gene ontologies between the IRE1α^KO^ signature high and low tertile patients. The count of genes enriching the term on the top x-axis is represented as a barplot, and the –log(10) *p* value for the terms on the bottom x-axis, represented as a black symbol. Exact *P* values are in the Source Data file. **d**–**n** Violin plots of xCell pipeline enrichment scores for microenvironment (**d** *P* < 0.0001), immune (**e** *P* < 0.0001), stromal (**f** *P* < 0.0001), DC (**g** *P* < 0.0001) cDC (**h** *P* < 0.0001), pDC (**i** *P* < 0.0001), CD8 + T cells (**j** *P* < 0.0001), Effector CD8 T cells (**k** *P* = 0.0011), CD4 T cells (**l** *P* = 0.0013), Effector CD4 T cells (**m** *P* = 0.0017), and Treg (**n** *P* < 0.0001) from the top and bottom tertiles of those with high (red, *n* = 169) or low (blue, *n* = 169) signature enrichment. Unpaired Student’s *t*-test, two-sided. *P < 0.05, **P < 0.001, and ***P < 0.0001. Source data are provided as a Source Data file.

Next, we determined if differentially expressed genes identified from RNA-seq analysis of IRE1α^WT^ vs. IRE1α^KO^ tumor cells harvested from HKP1 tumors could be exploited to develop an IRE1α-dependent gene signature that could predict outcome in human NSCLC. To this end, we systematically evaluated a variety of statistical parameters including fold-change (FC) and false discovery rate (FDR) to identify an optimal gene signature associated with survival in human NSCLC (Fig. 5a and Supplementary Table 3**)**. We posited that our pure mouse tumor cell signature could be applied to the TCGA-LUAD dataset, as each human tumor sample has a minimum of 80% cancer cells. We performed single sample gene set enrichment analysis (ssGSEA) on a discovery cohort of >300 LUADs available in the TCGA database^29^, ranking samples by their enrichment scores, comparing outcomes for the top and bottom tertiles for each signature, and evaluated for survival. We selected the log2FC >1 and FDR 1% gene signature (IRE1α^KO^ high comprised of 582 genes), as this appeared to comprise a robust number of genes for downstream analysis and provided marked survival benefits at both the quartile and tertile cutoff ranges (Fig. 5b). In a multivariate analysis, a Cox proportional-hazards regression model adjusted for clinicopathologic parameters (Age at time of diagnosis, gender, pathology, and TN stages) did not correlate OS with IRE1α signature (Supplementary Fig. 14a), and similarly, Multivariate Cox proportional-hazards regression models for IRE1α versus 1,000 random signatures of identical lengths showed specificity exceeding 82% for the IRE1α signature (Supplementary Fig. 14b).

Consistent with the HKP1 model, GO analysis (Log2FC >0.5, FDR <10%, *p* value < 0.05) highlighted alterations in immune-mediated processes in the IRE1α^KO^ upregulated signature human NSCLC cohort (Fig. 5c). GO analysis of the IRE1α^KO^ downregulated gene signature showed enrichment in mTORC1 signaling, hypoxia, cholesterol and steroid biosynthesis gene programs, and no lung cancer-specific modules were identified (Supplementary Fig. 13k).

To determine if the IRE1α^KO^ upregulated high and low signature group were associated with an altered immune landscape, as was observed in the IRE1α^KO^ murine tumors, we used the xCell pipeline^30^ to computationally estimate immune cell infiltration in the TME of the IRE1α^KO^ high signature patients from the top and bottom tertiles above. Patients enriched for IRE1α^KO^ high signature showed an increase in the microenvironment score (Fig. 5d), which is the sum of all immune and stromal cell scores (Fig. 5e, f), suggesting an overall enhanced immune milieu. Further evaluation of the xCell DC scores, showed that consistent with the murine data, there was enrichment of pan-DC (Fig. 5g), cDC (Fig. 5h), and plasmacytoid DC scores (Fig. 5i). Similarly, we observed enrichment in both CD8 and CD4 lymphocytes, and effector/memory T cells (Fig. 5j–m). Expanded analysis showed enrichment scores in additional lymphocytic populations (DCs, CD4, CD8, and gdT cells), and myeloid populations (Macrophages, neutrophils, and NK cells) in IRE1α^KO^ high signature patients (Supplementary Fig. 15). Compared to mouse tumors, IRE1α^KO^ high signature patients did not show an enrichment score in Foxp3 Tregs (Fig. 5n).

**Fig. 6:**
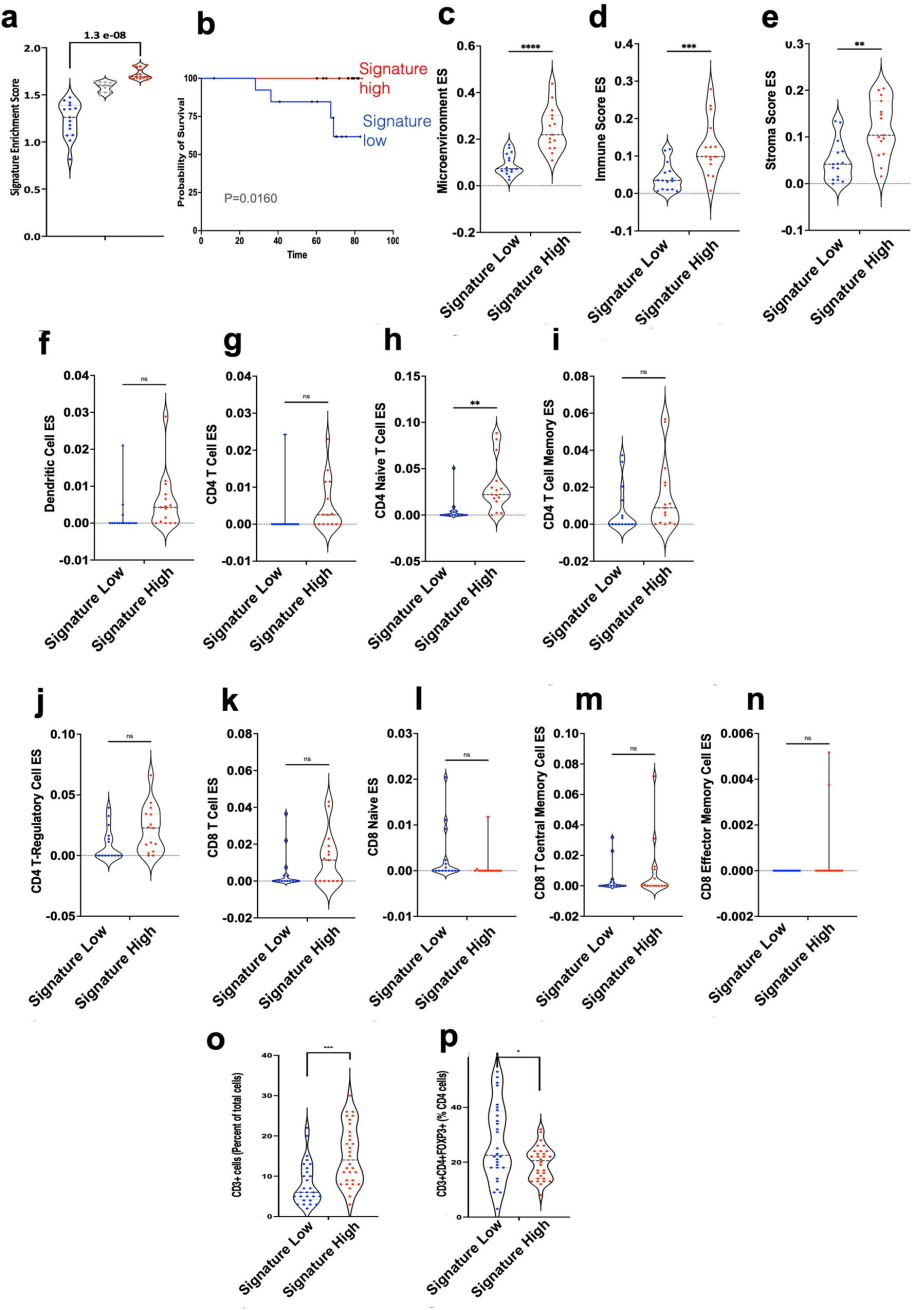
Validation of the IRE1a signature and RNA-seq deconvolution. **a** Violin plot of enrichment scores (ssGSEA) for IRE1α^KO^ gene signature (Log2 fold-change >1 and FDR 0.01) low (*n* = 15, blue), mid (gray, *n* = 14), and high (red, *n* = 15) NSCLC patients. One-way ANOVA with Tukey’s multiple comparisons test for ratios. **b** Kaplan–Meier survival plots depicting associations between overall survival (OS) and IRE1α^KO^ signature status of NSCLC patients stratified into the top and bottom tertiles (15 patients in each) for the IRE1a^KO^ gene signature. **c**–**n** Violin plots of xCell pipeline enrichment scores for microenvironment (**c** *P* < 0.0001), immune (**d** *P* = 0.001), stromal (**e** *P* = 0.001), dendritic (**f** *P* = ns), CD4 + T cells (**g** *P* = ns), CD4 + naïve (**h** *P* = 0.0027), CD4 memory (**i** *P* = ns), T-regulatory (**j** *P* = NS), CD8 + T cell (**k** *P* = ns), CD8 + Naïve (**l** *P* = ns), CD8 + TCM (**m** *P* = ns), and CD8 + effector memory (**n** P = ns) from the validation set of lung cancer patients from the IRE1α signature high (red, *n* = 15) and low (blue, *n* = 15) groups for the enrichment of various immune cells. Unpaired, two-tailed, Student’s *t*-test. *P < 0.05, **P < 0.001, and ***P < 0.0001. **o**, **p** IF analysis showing quantitation of CD3T cells (left panel) and Tregs (right panel) in tumor nests of validation set of lung cancer patients from the IRE1α signature high (red, *n* = 30) and low (blue, *n* = 30) groups. Unpaired, two-tailed, Student’s *t*-test. *P < 0.05, **P < 0.001, and ***P < 0.0001. Source data are provided as a Source Data file.

To validate the findings from the analysis of the TCGA datasets, we applied the murine IRE1α gene signature to an independent collection of 44 human lung tumors (Fig. 6a), which we had recently reported^31^. Consistent with the TCGA analysis, patients enriched for the IRE1α gene signature exhibited increased survival (Fig. 6b). Deconvolution of RNA-seq dataset from these patients showed that IRE1α^KO^ high signature patients exhibited an increase in microenvironment score, immune and stromal enrichment scores, together with enrichment of pan-DC, cDC, and plasmacytoid DC, CD8 and CD4 lymphocytes and effector/memory T cells (Fig. 6 c–n). To experimentally validate the computational deconvolution data, we used IHC, and observed increased infiltration of T cell lymphocytes, associated with a concomitant reduction in Tregs (Fig. 6 o, p). Together, these findings suggest that the IRE1α signature is associated with altered immune landscape and predicts outcomes in human NSCLC.

## Discussion

By developing a computational method to identify and quantitate the IRE1a-generated *XBP1s* isoform in public RNA-seq datasets, we identified that levels of this transcript are associated with poor overall survival in human NSCLC. Specificity was further assessed using a multivariate analysis model adjusted for age at the time of diagnosis, gender, pathology, and TN stages did not show a significant correlation between *XP1s* and survival. Our findings are consistent with prior reports indicating that increased XBP1s protein levels can predict NSCLC aggressiveness^32^, and that IRE1α is prognostic of recurrence in resected NSCLC patients^33^. Yet, whether this arm of the UPR promoted NSCLC progression and immunosuppression was uncertain. Through genetic ablation of IRE1α in mouse models of NSCLC, we uncovered a functional role of IRE1α activation in accelerating malignant progression, leading to poor host survival. Remarkably, cancer cell-intrinsic loss of IRE1α provoked potent antitumor immune responses by altering both the lymphoid and myeloid cell subsets in the TME, a new paradigm in NSCLC, consistent with emerging evidence in melanoma and glioblastoma showing that cancer cell-intrinsic pathways alter the immune landscape in the TME^21–23^. A previous study on cancer intrinsic IRE1α-XBP1 signaling in breast cancer employed immunocompromised mice and therefore, did not evaluate the impact of XBP1 loss on the immune microenvironment^10^. In our study, Rag2 knockout mice failed to show tumor regression upon challenge with IRE1α^KO^ HKP1 tumors, though a trend towards extended survival was still evident in this genetic context. This result is likely due to improved NK cell function in IRE1α^KO^ tumors or by direct protumorigenic programs mediated by IRE1α cell-intrinsic signaling in the cancer cell^6^.

By curating an Immunomodulator dataset of known tumor-intrinsic signaling immunomodulatory pathways, and using Enricher, an unsupervised approach, we identified enrichment of the eicosanoid pathway following IRE1α loss. This finding highlighted that cancer cell-intrinsic IRE1α drove mPGES-1 expression, enabling the production of the bioactive lipid PGE_2_ in the TME. Of note, we have recently reported that the IRE1α-generated XBP1s transcription factor directly transactivates *COX2* and *PTGES* genes in human leukocytes to enable PGE_2_ production in the context of pain^28^. PGE_2_ is known to reprogram immune cells by virtue of promoting differentiation of Tregs^24^, enhancing MDSC function^25,26^, and blocking DC differentiation, infiltration, and activation^27^. Remarkably, ectopic expression of *Ptges* rescued IRE1α^KO^ loss-of-function phenotypes, establishing the role of tumor-intrinsic IRE1α-XBP1-PGE_2_ signaling in NSCLC. Restoring PGE_2_ expression in IRE1α KO cells resulted in a trend towards an increase in Tregs and a decrease in CD4 TNFα and CD8 TNFα cell as expected. However, this rescue did not reach statistical significance. Possibly, early and constitutive overexpression of mPEGS1 in IRE1α^KO^ tumor cell overrides the exquisite control exerted by the ER stress response mediated by the IRE1a-XBP1 pathway. As a consequence, constitutive mPEGS1 expression may have directly contributed to direct tumor growth without significantly affecting immune responses. Defining the precise immune cells in the TME that are direct targets of the IRE1α-XBP1-PGE_2_ pathway warrants further investigation.

RNA-seq analysis of IRE1α^WT^ vs. IRE1α^KO^ cancer cells identified a gene signature predictive of overall survival in patients with NSCLC. The specificity of the IRE1α signature was further assessed using a multivariate analysis model adjusted for clinicopathologic parameters including age at the time of diagnosis, gender, pathology and TN stages, which did not correlate OS with the IRE1α signature. Similarly, the evaluation of the IRE1α signature versus 1000 random signatures of identical lengths showed specificity exceeding 82% for the IRE1α signature. Furthermore, RNA-seq deconvolution showed that NSCLC patients enriched for IRE1α signature showed an increase in both the Immune and Stromal enrichment scores suggesting an overall enhanced immune milieu, consistent with the murine data. The findings from the TCGA analysis were validated in an independent cohort of human lung tumors both in the context of the immune milieu and survival outcomes. While the human TCGA RNA-seq deconvolution data did not show significant reduction in Tregs in signature high patients, deconvolution of the validation cohort showed a reduction in Tregs in signature high patients, a finding confirmed by IF analysis. Given that TCGA samples constitute a minimum of 60–80% cancer cells^34^, it is possible that may not have sufficient numbers of some immune subsets including Tregs. In contrast, percent tumor purity was not used as a criteria in the validation set.

Our study demonstrating that cancer cell-intrinsic activation of IRE1α signaling constitutes a driver of immunosuppression in the TME, suggests that targeting this ER stress sensor may represent a “two-pronged” therapeutic approach to restrain malignant cells, while concomitantly eliciting potent anti-cancer immunity. We observed that pharmacological inhibition with MKC8866 an inhibitor of IRE1α endoribonuclease^35^ significantly reduced levels of *Xbp1s* and its canonical targets in the lung, however, treatment of HKP1-bearing mice failed to control tumor progression (data not shown). Possibly, the IRE1α-XBP1 pathway has been shown to be simultaneously activated in multiple tumor-infiltrating immune cells, including dendritic cells^6^, MDSCs^36^, Macrophages^37^, T cells^12^, and NK cells^38^, therefore, it is likely that global IRE1α inhibition in NSCLC-bearing mice may induce confounding effects on overall antitumor immunity and disease progression. Therefore, our observations underscore the importance of targeting cancer cell-specific IRE1α in the specific setting of NSCLC. Indeed, efforts to develop pharmacological inhibitors that selectively target IRE1/XBP1s in malignant cells are underway^39–41^, and these inhibitors may have potential clinical utility. Furthermore, using IRE1α -mediated differentially regulated gene networks, we developed a murine IRE1α gene signature prognostic of survival in NSCLC patients. This IRE1α-dependent gene signature may enable the identification of patients that might benefit from treatment with IRE1α inhibitors. Additionally, the IRE1α gene signature also has the potential to serve as a prognostic/diagnostic biomarker of the disease and may allow monitoring efficacy of therapies targeting the IRE1α-XBP1 pathway.

## Methods

### Human *XBP1* splice variant quantification and signature enrichment

The human lung adenocarcinoma (LUAD) RNA-seq dataset was downloaded from the NCI Genomics Database Commons (GDC)^42^. Samples were aligned to Hg19 using Bowtie2. To align short reads to the *XBP1* unspliced transcript, we employed parameters to depenalize long gaps, allowing us to estimate the fraction of spliced reads over read coverage. Using Karkkainen’s blockwise algorithm-based bowtie2-build (version 2.3.0), the index files for the gene sequence were generated respectively. The alignment was then performed using bowtie2 aligner, with parameters optimized to make it more sensitive. Some of the significant adjustments were made to parameters such as *-D* adjusted to a higher number, in order to increase the number of total tries for aligning a given read until a best or second-best alignment is found. Also, instead of local alignment, *end-to-end* alignment was specified for a relatively lenient alignment. Read gap *-rdg* and reference gap *-rfg* length penalties were relatively decreased and disallowed gaps at the start or end of reads *-gbar* were increased. Each of the parameters were tested with combinations configured in increments on validation RNA-Seq data, prior to establishing the selected ones. Below are values for all specified parameters.

bowtie2 -p 8 -D 20 -R 3 -N 0 -L 20 -i S,1,0.50 -end-to-end -rdg 1,1 -rfg 1,1 -gbar 10

The aligned samples are then processed using SAMtools mpileup (version 1.2) to call for base information and indel calling against the* XBP1* gene sequence reference. The parameters for calling indels were adjusted to detect the specific 26 NT indel with regards to data coverage, base qualities, alignment mapping quality and enhancing indel calling capacity. Parameters -m 3 -L 50000 -F 0.0002 were set in order to increase the chances of finding at least three supporting reads with relatively low frequency and increasing max depth before skipping calling an indel. Error probability on scaled gap extensions -e 15 and -o 20 gap open sequencing error probabilities were reduced from the default value to increase the frequency of detecting longer indels, increasing overall indel detection capability.

The called base changes were then processed using bcftools (from SAMtools version 1.14) view to convert from the pileup format to a VCF file. An in-house script was then used to parse through all VCFs for each sample, to filter in indel statistics with the specific *XBP1s* sequence, if detected. Next, to obtain the relative percentage of *XBP1s* the quantified indel coverage listed as QC passed non-reference base read counts (number of spliced reads) were normalized to the reference reads at the detected indel position column listed as Q13 reference base counts. The spliced transcripts quantified are then converted as the ratio of the reference XBP1 to obtain the XBP1s splicing percentage Below are values for all specified parameters used for indel calling.

### Commands

samtools-1.2/samtools mpileup -m 3 -L 50000 -e 15 -o 20 -F 0.0002 -r “NM_005080”

-u -f *XBP1*.fa aligned_sorted.bam| bcftools view - > OUTPUT_XBP1.INDELS

Patients were subsequently ranked by the percent of the spliced *XBP1* isoform, and the top and bottom tertiles were evaluated for survival and association with any other clinicopathologic descriptors. Cufflinks was used to measure transcript abundances in Fragments Per Kilobase of exon model per million mapped reads (FPKM) with upper-quartile normalization and sequence-specific bias correction. Global UPR and individual branch activation between the *XBP1* high and *XBP1* low groups were evaluated via gene set enrichment analysis (GSEA, version 4.2.2). The Unfolded Protein Response signature was collected from the Hallmark collection from the Molecular Signatures Database (MSigDB), and the ATF6, PERK, and XBP1 downstream target signatures were collected from the Harmonizome database^43^.

### Cell culture, mouse models, tumor growth, and imaging

All animal work was performed in accordance with protocols approved by the Institutional Animal Care and Use Committee. Two murine orthotopic NSCLC tumors HKP1 (*Kras*^*G12D*^*p53*^−*/*−^)^16^ and CMT-167 expressing mCherry-Luciferase were employed in this study. Cells were maintained in DMEM supplemented with 10% FBS, 1% Penicillin/Streptomycin, and 1% l-Glutamine. To generate lung tumors in mice, 150,000 HKP1 or 100,000 CMT-167 cells were r injected via tail vein into 8-week-old syngeneic immunocompetent female C57BL/6J (Stock # 000664) or RAG2 deficient (stock # 008449) mice purchased from the Jackson Laboratory (Bar Harbor, Maine). Tumor growth in vivo was measured via bi-weekly bioluminescence imaging (BLI). Briefly, mice were anaesthetized with isofluorane, prior to a retro-orbital injection of 75 mg/kg d-luciferin (Promega). BLI was measured by placing the mice into the Xenogen IVIS system in a supine position and measuring photon flux in the Living Image software suite (Living Image, Xenogen). In brief, the same circular region of interest (ROI), encompassing the thorax of the mouse, was placed on each mouse and the photon flux was measured. These values were employed to generate all BLI plots. Given that tumors were orthotopic to the lungs, BLI measurements were used to monitor tumor growth. Animals were monitored for any signs of stress, pain, or discomfort, and if such a symptom was observed appropriate intervention strategies were implemented including the administration of analgesics, anesthetics, and/or euthanasia. In a situation where the discomfort resulted from tumor burden, mice were sacrificed prior to or immediately upon observation of discomfort.

### mRNA extraction, reverse transcription, and quantitative RT-PCR

Total RNA was isolated from cell lines, bulk tissues and sorted cell populations as described in the results section by using the RNeasy kit (Qiagen) and performing on-column DNAse I digestion (Qiagen) per the manufacturer’s protocol. About 250 ng of mRNA was reverse transcribed using qScript cDNA SuperMix (Quanta bio). qRT-PCR was performed using SsoAdvanced Universal SYBR Green Supermix (Bio-Rad). All samples were run on a Bio-Rad CFX96 Real-Time System (Bio-Rad), in triplicate with melt-curve analysis. The relative abundance of target genes was compared relative to the housekeeping gene β-actin by the 2^−dCt^ method. A list of primers are shown (Supplementary Table 4).

### Generation of knockout cell lines

CRISPR knockouts in the HKP1 and CMT-167 cell lines were generated using sgRNA-CAS9 ribonucleoprotein (RNP) complexes being electroporated using the NEON transfection system (Thermo Fisher). Materials used to generate sgRNA-CAS9 RNP complexes were purchased from Integrated DNA Technologies. In brief, duplexed sgRNAs were generated by mixing ATTO-550-tracrRNA and target crRNAs in a 1:1 ratio, heating the mixture at 95 °C for 5 min. RNPs were generated by mixing sgRNAs with CAS9 in a 1:1.2 ratio (CAS9:sgRNA) and incubating for 20 min at room temperature. About 500,000 cells were electroporated at a time using the NEON transfection system with electroporation enhancer, RNP and cells. Cells were administered 2 pulses, spaced 30 ms apart, at 1200 V. Electroporated cells were then transferred into six-well plates with normal media and 24 h later ATTO-550+ cells were sorted into single wells on a 96-well plate. Single colonies were grown out and screened to evaluate the IRE1α^KO^.

CRISPR gene editing was evaluated by sanger sequencing and gDNA was extracted using the DNeasy kit, following manufacturers’ protocols. A 100 bp region flanking the CRISPR target sequence was amplified by PCR using iProof HF master max (Bio-Rad). Samples were then run on a 2% agarose gel and gel purified using the QIAquick gel extraction kit (Qiagen). gDNA content was measured by nanodrop and 100 ng was sent for Sanger sequencing at Macrogen. Sequencing files were aligned using DNASTAR Lasergene 17 SeqMan Pro. List of primer and guide sequences are shown (Supplementary Table 4).

### Western blot

Lysates from HKP1 lung tumor cells were prepared using 20 mM Tris, pH 8, 135 mM NaCl, 1% NP-40, 10% glycerol, 1 mM PMSF, and a complete protease inhibitor cocktail (Roche) lysis buffer. About 25 μg of protein lysate was loaded into 4–15% gradient SDS-polyacrylamide gels (Bio-Rad) and transferred to PVDF membranes. Membranes were subsequently blocked with 5% milk and probed with the indicated primary antibodies: IRE1α (Cell Signaling), XBP1s (Biolegend), and α-Tubulin (Proteintech). Membranes were then incubated with a peroxidase-conjugated correspondent secondary antibody (R&D Systems) and detected using the ECL prime western blotting system (Amersham) on Hyblot CL Autoradiography film (Denville Scientific Inc.). List of antibodies are shown (Supplementary Table 5).

### Flow cytometry for immunophenotyping

At day 10 and day 14, mice were euthanized, lungs were perfused with HBSS, dissected, separated from the heart and thymus, minced, and ground through a 140-μm wire mesh (Cell Screen/ 100 mesh, Bellco Glass, Inc.) with a glass pestle, into RPMI-1640. Single-cell suspensions were generated by passing the samples through a 70 μm cell strainer (Cell Strainer, Nylon; Falcon). Cells were then stained following the standard flow cytometry protocol^44^. In brief, cells were first stained with Zombie Aqua Fixable Viability dye in accordance with the manufacturer’s protocol. Following this, cells receiving surface stains were FC blocked (CD16/32, 1:100 BD Biosciences), incubated with primary antibodies, and fixed with 1% formaldehyde, and resuspended in FACS Buffer. Samples were covered in aluminum foil and stored at 4 °C until analysis.

For intracellular staining, if stimulation and golgi blocking were required, samples were incubated for 4 h in complete RPMI at 37 °C in a humidified incubator, with PMA (100 ng/mL), ionomycin (1 μg/mL), Brefeldin A (Biolegend), and Monensin (Biolegend). Following this, samples were surface stained as above before undergoing fixation and permeabilization (eBioscience) in accordance with the manufacturers’ protocol. Following this samples were stained with intracellular antibodies, washed and resuspended in FACS Buffer. Samples were covered in aluminum foil and stored at 4 °C until analysis (less than 24 h later). Data were acquired on a Becton-Dickinson LSR II and analyzed with FlowJo 10 (Version 10.8.1). List of antibodies (Supplementary Table 5).

### RNA-seq and data analysis

Single-cell suspensions were generated and stained with CD45, and Epcam, as above without fixation. Viable CD45− mCherry+ Epcam+ cells were sorted into Trizol. mRNA was extracted as previously described. mRNA concentration was then evaluated by Nanodrop (Thermo Fisher), and the quality of the mRNA was evaluated by Bioanalyzer (Agilent).

cDNA libraries were generated using the Illumina TruSeq RNA Sample Preparation kit V2 with non-stranded Poly A selection, in accordance with the manufacturer’s protocol. About 2 × 50 bp single-end sequencing was performed on the HiSeq 4000 sequencer. Raw sequencing reads were aligned to the mm9 mouse reference genome using Tophat2 (version 2.0.1). Cufflinks (version 2.2.1) was used to measure transcript abundances in Fragments per Kilobase of exon model per Million mapped reads (FPKM), with upper-quartile normalization and sequence-specific bias correction and non-normalized raw counts.

FPKM expression matrices were employed for heatmaps and Log2 transformed FPKM values were used for principal component analysis and visualized using ggplot2 in R (version 3.3.3). Heatmaps were made using the Pheatmap (version 1.0.12) and RColorBrewer (version 1.1-2) packages, and the Venny web portal (version 2.1) was used to make all Venn diagrams. Differential gene expression (DGE) was performed on non-normalized counts using the standard DESeq2 package (version 1.28.1) protocol in R, with pairwise comparisons of all WT vs KO, day 10 WT vs KO, and day 14 WT vs KO. Significance cutoff values were set at log2 fold-change > 0.5, *p* value <0.05 and false discovery rate <10%. Pathway and ontology enrichment was performed using the Enrichr web portal. Enrichment of specific signatures was performed by using GSEA as described above.

NSCLC-specific IRE1α gene expression signatures were generated by taking the up and downregulated genes identified by DGE at a variety of cutoffs and applying them to the TCGA-LUAD mRNA-seq dataset using the ssGSEA function of the GSVA package (version 1.36.3), generating a per patient enrichment score for each signature. Patients were ranked by enrichment score and evaluated for clinicopathologic factors in the top and bottom tertiles. Immune cell deconvolution scores were generated by running TCGA mRNA expression data through the xCell pipeline.

### Cell cycle, proliferation, apoptosis, and invasion measurements

The cell cycle was measured through the Click-iT Edu (Thermo Fisher Scientific) system in accordance with the manufacturer’s protocol. 10^5^ IRE1α^WT^ or IRE1α^KO^ HKP1 cells were plated in a six-well plate and allowed to settle overnight, cells were then serum starved for 12 h, prior to evaluating the cell cycle. Cells were then incubated with a 1:1000 dilution of Click-iT Edu for 30 min at 37 °C. Cells and subsequently stained with FxCycle Violet (Thermo Fisher Scientific) and evaluated on a flow cytometer.

Longitudinal cell proliferation was accomplished by plating, 5 × 10^3^ IRE1α^WT^, IRE1α^KO^, or parental HKP1 cells per well were plated in a 96-well plate, cells were plated for measurement at 0, 24, 48, and 72 h with all conditions in triplicate. Cells were allowed to settle overnight, and serum starved for 12 h before measurement of the 0 h timepoint. Proliferation was then measured by using the MTT assay (Promega) performed in accordance with the manufacturer’s protocols, with luminescence measured using a luminometer.

Apoptosis was measured by plating 10^5^ IRE1α^WT^ or IRE1α^KO^ HKP1 cells in a six-well plate and allowing them to settle overnight, cells were then serum starved for 12 h. Cells were collected and stained with FITC conjugated Annexin V (Apoptosis detection kit, Biolegend) and 7-aminoactinomycin D (Thermo Fischer Scientific) and analyzed by LSR II flow cytometry and Flowjo 10 (Flowjo).

The invasion was evaluated by plating 10^6^ IRE1α^WT^ or IRE1α^KO^ HKP1 cells in a 10 cm^2^ plate and allowing them to settle overnight, cells were then serum starved for 12 h prior to initiating the experiment. About 10^4^ IRE1α^WT^ or IRE1α^KO^ cells were resuspended in 500 μL of serum-free DMEM media and added to the inside of Matrigel inserts (BD BioCoat), DMEM complete media with 10% serum was added to the outside of inserts. Chambers were incubated for 24 h at 37 °C. The non-invading cells were then removed by scrubbing the surface with a cotton-tipped swab. After 24 h, invasion chambers were removed and stained using Kwik-Diff Stains according to the manufacturer’s instructions (Thermo Fisher Scientific). Six reference points were randomly selected from each well and the number of invading cells were normalized to control.

### Collected immunomodulator dataset

To generate a database of cancer cell-intrinsic signaling pathways that directly reprogram the tumor immune microenvironment, we manually curated information from Pubmed, abstracts, and databases using search terms described in Supplementary Data 3. Studies were also screened to evaluate whether the signaling was tumor cell-specific, or derived from bulk tissues/infiltrating immune cells, only the former were included for further evaluation. For molecules secreted by tumor cells (e.g., cytokines and chemokines) the validated upstream signaling mechanism was included as components of the signature, for receptors the downstream target genes, partners and secreted molecules were included. Oncogenic drivers with validated specific downstream targets were included as a specific mechanism and not all known differentially expressed genes resulting from their knockout/expression. Additionally, in the event multiple studies focused on a specific pathway, e.g., p53, if p53 were studied alone it would be included as part of a single term, however, in the context of p53 and a second alteration, such as KRAS, they would instead form a new term. A minimum size cutoff of five genes was applied to any potentially included study. The pathway name, genes implicated, whether they were immune suppressive or activating, species, organ and reference were recorded. We then intersected these lists, with the differentially regulated genes, and for any highlighted genes identified how many genes from that signature were enriched relative to the total interested gene list. Highlighted genes were heatmap, and the parts of the whole were visualized as a pie chart.

### cDNA overexpression rescue vector construction and packaging

Ptges1 cDNA was generated by isolating mRNA from the parental HKP1 cell line, and reverse transcribing it to cDNA using qScript cDNA SuperMix. Forward and reverse primers with appropriate overhangs were designed to amplify the Ptges1 cDNA. The amplified cDNA product was gel purified, and Sanger sequencing was used to confirm the identity of the product. The cDNA was subsequently inserted into the pCDH-CMV-MCS-EF1α-GFP-T2A-Puro (pCDH, System Biosciences, Cat. # CD513B-1) lentiviral vector at the NheI and NotI restriction sites, in accordance with the manufacturer’s protocol. Lentivirus was generated by packaging the validated pCDH Ptges1 plasmid using the VSVG psPAX2 system, and transfecting HEK293T cells using lipofectamine 3000 (Invitrogen), in accordance with the manufacturers standard protocol. List of primer and guide sequences (Supplementary Table 4).

### Survival analysis

Patients were ranked by the percent of the spliced XBP or high and low IRE1α signature groups and the top and bottom tertiles (one-third) were evaluated for survival. Multivariate survival analysis was performed using the Cox proportional-hazards additive regression model between the high and the low groups, where the low group was the reference group. The model was adjusted for age at diagnosis, gender, pathology, TN stages and smoking history.

### Random signature generation

541 out of the 582 genes in the IRE1α signature had human orthologs in the expression profiles obtained for the LUAD-TCGA cohort. After excluding the genes from the IRE1α signature, 100 random signatures of length 541 were sampled from the protein-coding genes (*n* = 19,171). These random signatures were scored in the LUAD cohort using ssGSEA, similar to what was done for the IRE1α signature score estimation. Multivariate survival analysis was performed between the tumors grouped by the top and bottom 1/3rd of the enrichment score distribution (see Survival analysis section above for details).

To validate the findings from the analysis of the TCGA datasets, the murine IRE1α gene signature was applied to an independent collection of 44 human lung tumors previously reported^31^. The was approved by the New York-Presbyterian-Weill Cornell Medicine institutional review board.

### Statistical analysis

Unless noted otherwise, all experiments were repeated at least two times and results were similar between repeats. All statistical analyses were done using Graph Pad Prism 9.2.0. Differences between the means of experimental groups were calculated using, when only two groups were analyzed, a Student’s *t*-test was applied, one-way ANOVA with Tukey’s multiple comparisons test was used ratios, and two-way ANOVAs with Tukey’s multiple comparisons test was used for all other analyses. Error bars represent SEM from independent samples assayed within the represented experiments. Survival rates were compared using the log-rank Mantel-Cox test. All survival experiments used at least six mice per group. This number provides a 5% significance level and 95% power to detect differences in survival of 20% or greater.

### Reporting summary

Further information on research design is available in the Nature Portfolio Reporting Summary linked to this article.

## Supplementary information

Supplementary Information

Description of Additional Supplementary Files

Supplementary Data 1

Supplementary Data 2

Supplementary Data 3

Reporting Summary

## Data Availability

The publicly available LUAD data used in this study are collected from TCGA [https://portal.gdc.cancer.gov/projects/TCGA-LUAD]. The RNA-Seq data generated in this study have been deposited in the GEO database under accession code GSE202939. The murine IRE1α gene signature was applied to an independent collection of 44 human lung tumors previously reported^31^. These sequencing data were not generated for the purpose of this study but can be obtained upon request from: [https://www.ncbi.nlm.nih.gov/projects/gap/cgi-bin/study.cgi?study_id=phs002818.v1.p1]. The remaining data were available within the Article, Supplementary Information, or Source Data file. Source data are provided with this paper.

## Source data

Source Data

## Code availability

The pipeline to detect Spliced XBP1 from Bulk RNA-Sequencing Data is available at: [https://github.com/akv3001/RNASeq-Based-XBP1s-Detection-.git].
