# Supplementary material for: Tumor-intrinsic IRE1α signaling controls protective immunity in lung cancer
Source: Nat Commun. 2023 Jan 9;14:120. doi: 10.1038/s41467-022-35584-9 (PMC9829901; doi:10.1038/s41467-022-35584-9)
Supplement: Supplementary file 1 — Supplementary Information [file 41467_2022_35584_MOESM1_ESM.pdf]

Supplementary Information for

Tumor-intrinsic IRE1α signaling controls protective immunity in lung cancer.

Crowley et al.

Supplementary Fig. 1 is related to Fig. 1

Supplementary Fig. 2 is related to Fig. 2
Supplementary Fig. 3 is related to Fig. 2
Supplementary Fig. 4 is related to Fig. 2
Supplementary Fig. 5 is related to Fig. 2
Supplementary Fig. 6 is related to Fig. 2

Supplementary Fig. 7 is related to Fig. 3
Supplementary Fig. 8 is related to Fig. 3
Supplementary Fig. 9 is related to Fig. 3
Supplementary Fig. 10 is related to Fig. 3

Supplementary Fig. 11 is related to Fig. 4
Supplementary Fig. 12 is related to Fig. 4
Supplementary Fig. 13 is related to Fig. 4

Supplementary Fig. 14 is related to Fig. 5

Supplementary Fig. 15 is related to Fig. 6

Supplementary Data 1 is related to Fig. 3
Supplementary Data 2 is related to Fig. 3
Supplementary Data 3 is related to Fig. 4

Supplementary Table 1 is related to Fig. 1
Supplementary Table 2 is related to Fig. 1
Supplementary Table 3 is related to Fig. 5
Supplementary Table 4. For all Figs.
Supplementary Table 5. For all Figs.

Supplementary Fig. 1

a Human (hg19)

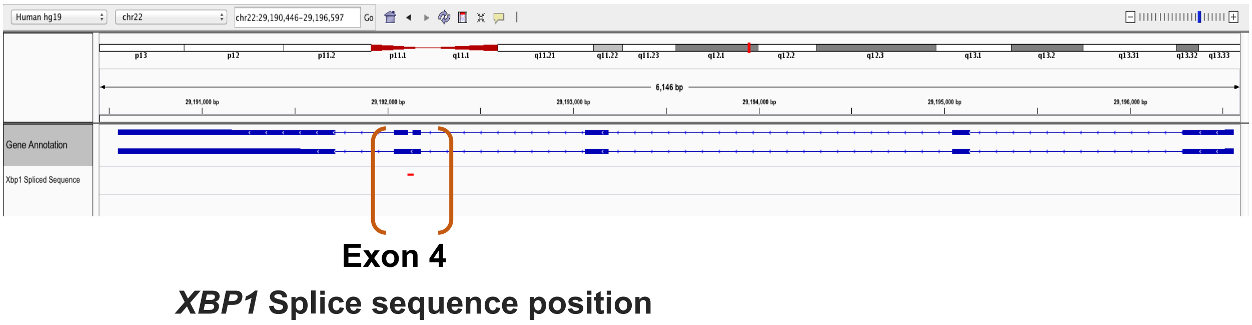

b

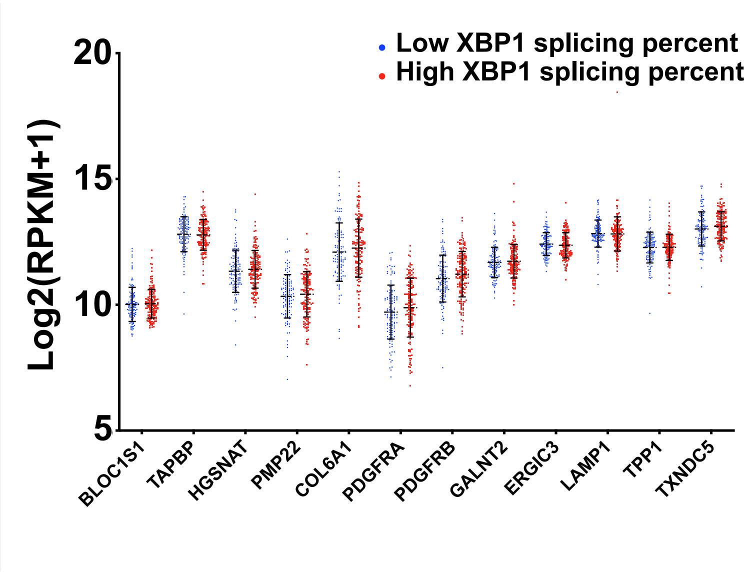

Supplementary Fig. 1. XBP1s pipeline, multivariate analysis and expression of RIDD targets in human TCGA LUAD.

a, Location of *XBP1* spliced sequence position in Exon 2.

b, Log_2 RPKM expression values of RIDD targets genes in *XBP1s* low (blue, n=103) and high (red, n=103) TCGA LUAD patients. Data are represented as mean ± SD.

Supplementary Fig. 2

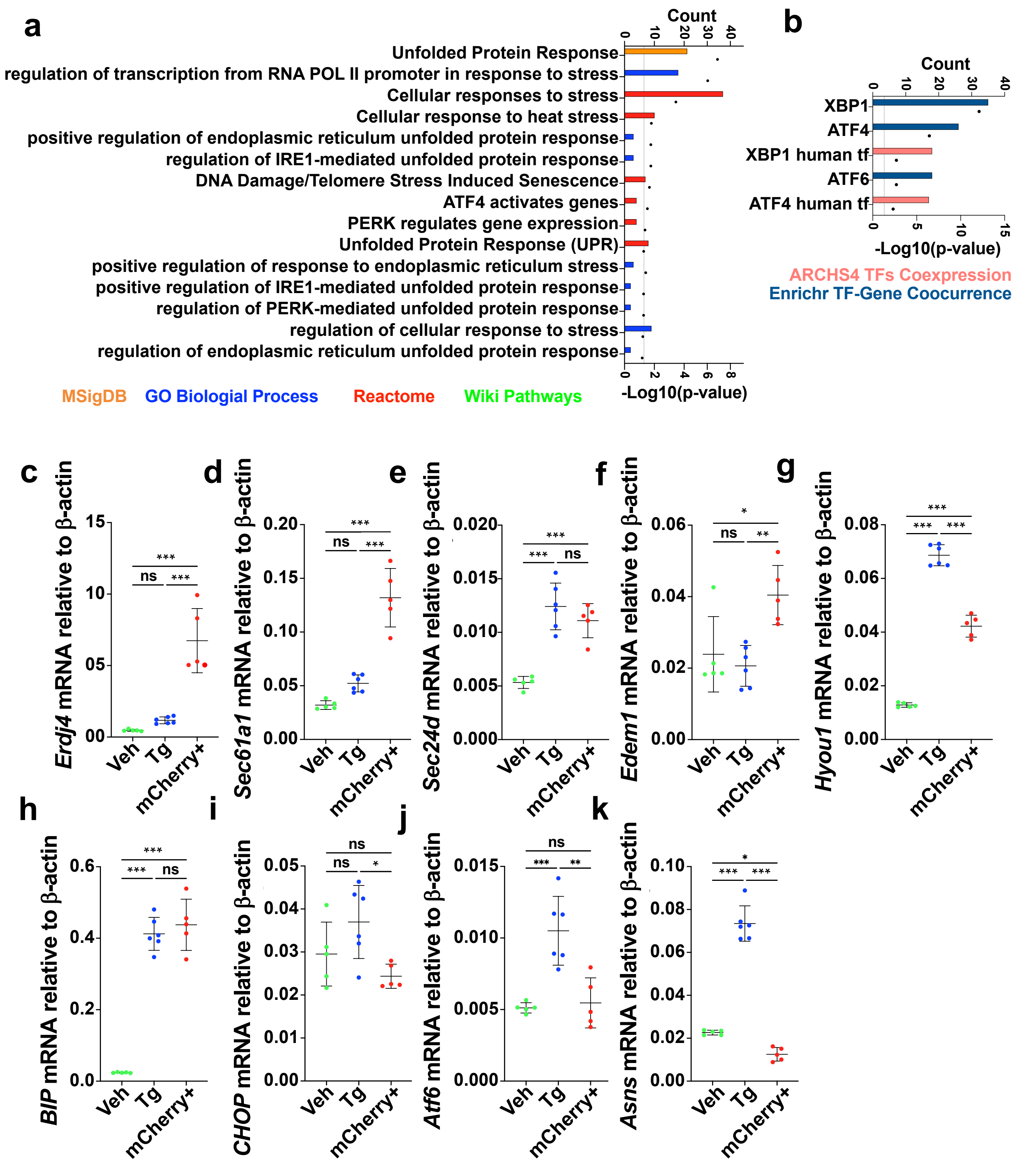

Supplementary Fig. 2. IRE1α-XBP1 signaling is activated in HKP1 tumor cells.

a, Top 15 upregulated terms enriched in HKP1 vs normal lung epithelial cells from MSigDB (orange), GO Biological Process (Blue), Reactome (Red) and Wiki Pathways (Green). The count of genes is on the upper y-axis represented as a barplot, and the –log10p-value is plotted on the lower y-axis, represented as a dot above its corresponding bar.

b, Top 5 transcription factors enriched in HKP1 vs normal lung epithelial cells from the ARCHS4 (pink), and EnrichR transcription factor co-occurrence (teal) databases. The count of genes is on the upper y-axis represented as a barplot, and the –log10p-value is plotted on the lower y-axis, represented as a dot above its corresponding bar.

c-k, RT-PCR of XBP1s target genes (Erdj4 (**c,** Veh vs Tg, P =NS, Veh vs mCherry P<0.0001, Tg vs mCherry, P <0.0001), Sec61a1 (**d,** Veh vs Tg, P = NS, Veh vs mCherry, P<0.0001, Tg vs mCherry, P<0.0001), Sec24d (**e,** Veh vs Tg, P<0.0001, Veh vs mCherry p<0.0001, Tg vs mCherry, P= NS), Edem1 (**f,** Veh vs Tg p = NS, Veh vs mCherry, P = 0.0186, Tg vs mCherry P=0.0043), & Hyou1 (**g,** Veh vs Tg p<0.0001, Veh vs mCherry p<0.0001, Tg vs mCherry, P<0.0001)) and global UPR markers (BIP (**h,** Veh vs Tg p<0.0001, Veh vs mCherry, P<0.0001, Tg vs mCherry p = NS), CHOP (**i**, Veh vs Tg, P = NS, Veh vs mCherry, P = NS, Tg vs mCherry, P= 0.0243), Atf6 (**j,** Veh vs Tg, P= 0.0007, Veh vs mCherry, P= NS, Tg vs mCherry, P= 0.0012), and Asns (**k,** Veh vs Tg, P<0.0001, Veh vs mCherry, P = 0.0285, Tg vs mCherry, P<0.0001)) in vehicle or Tg (n = 5 each) treated HKP1 cells *in vitro* or mCherry+ cells harvested from HKP1 tumors *in vivo* (n = 5). One-way ANOVA with Tukey's multiple comparisons test for ratios; $^{*}P < 0.05$, $^{**}P < 0.001$, $^{***}P < 0.0001$.

Supplementary Fig. 3

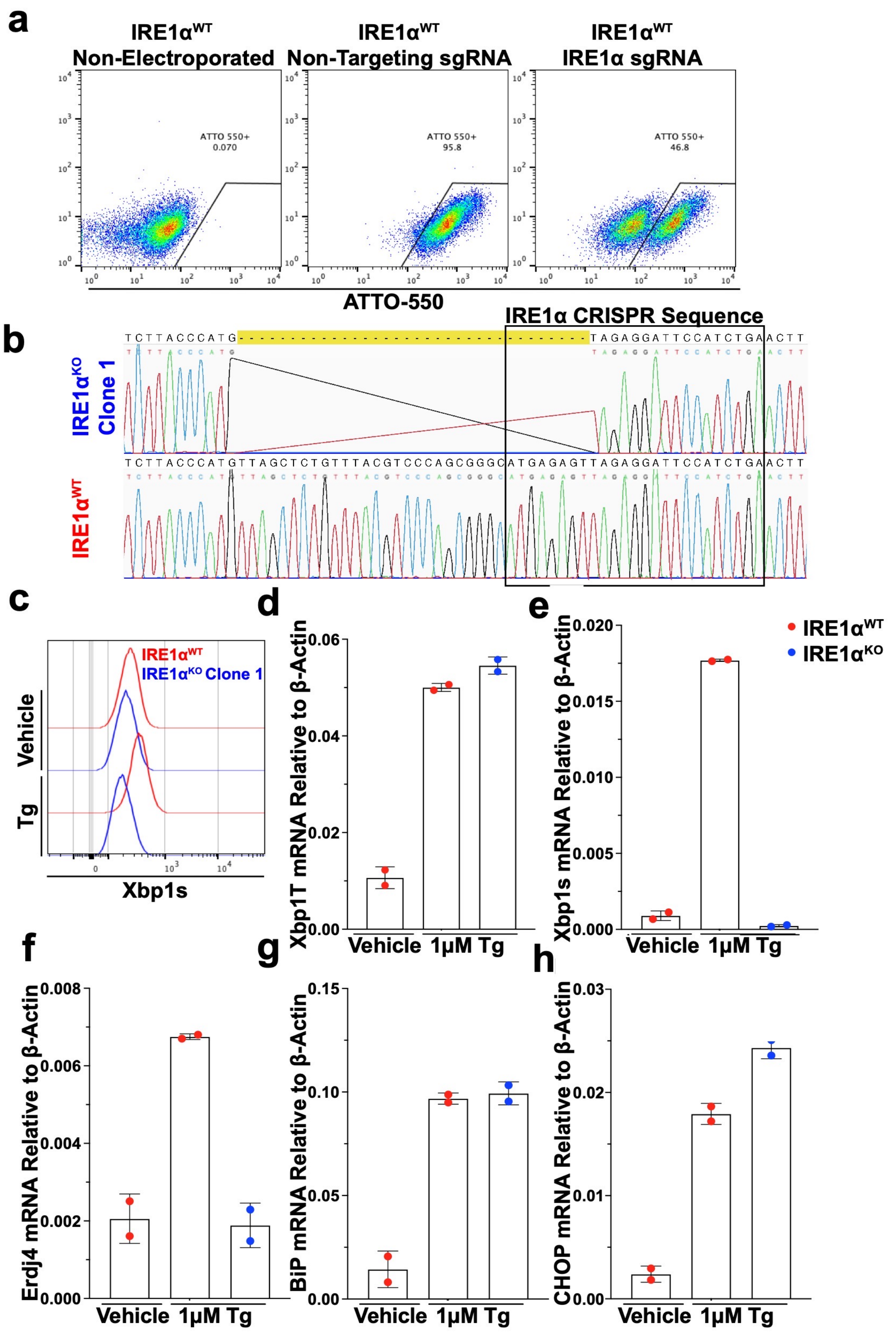

Supplementary Fig. 3. Generation and screening of IRE1α^{KO} HKP1 clones.

a, Representative flow cytometry plot showing gating of NEON-electroporated HKP1 cells with fluorescently labeled ATTO-550 Tracr-RNA.

b, Fluorogram representation of Sanger sequencing of the IRE1α CRISPR target sequence (black box), for an *IRE1α*^{WT} (red) and *IRE1α*^{KO} (blue) single colony clone.

c, Sample flow cytometry histogram of XBP1s protein expression, in Vehicle vs Thapsigargin treated IRE1α^{WT} (red) and *IRE1α*^{KO} (blue) single colony clones.

d-h, Barplot of qPCR of Vehicle (n=2) vs Thapsigargin (n=2) treated IRE1α^{WT} (red) or *IRE1α*^{KO} (blue) HKP1 cells for *total Xbp1* (**d**), *Xbp1s* (**e**), *Erdj4* (**f**), and non-IRE1/XBP1 targets *BiP* (**g**) and *CHOP* (**h**). Data are shown as mean ± SD.

Supplementary Fig. 4

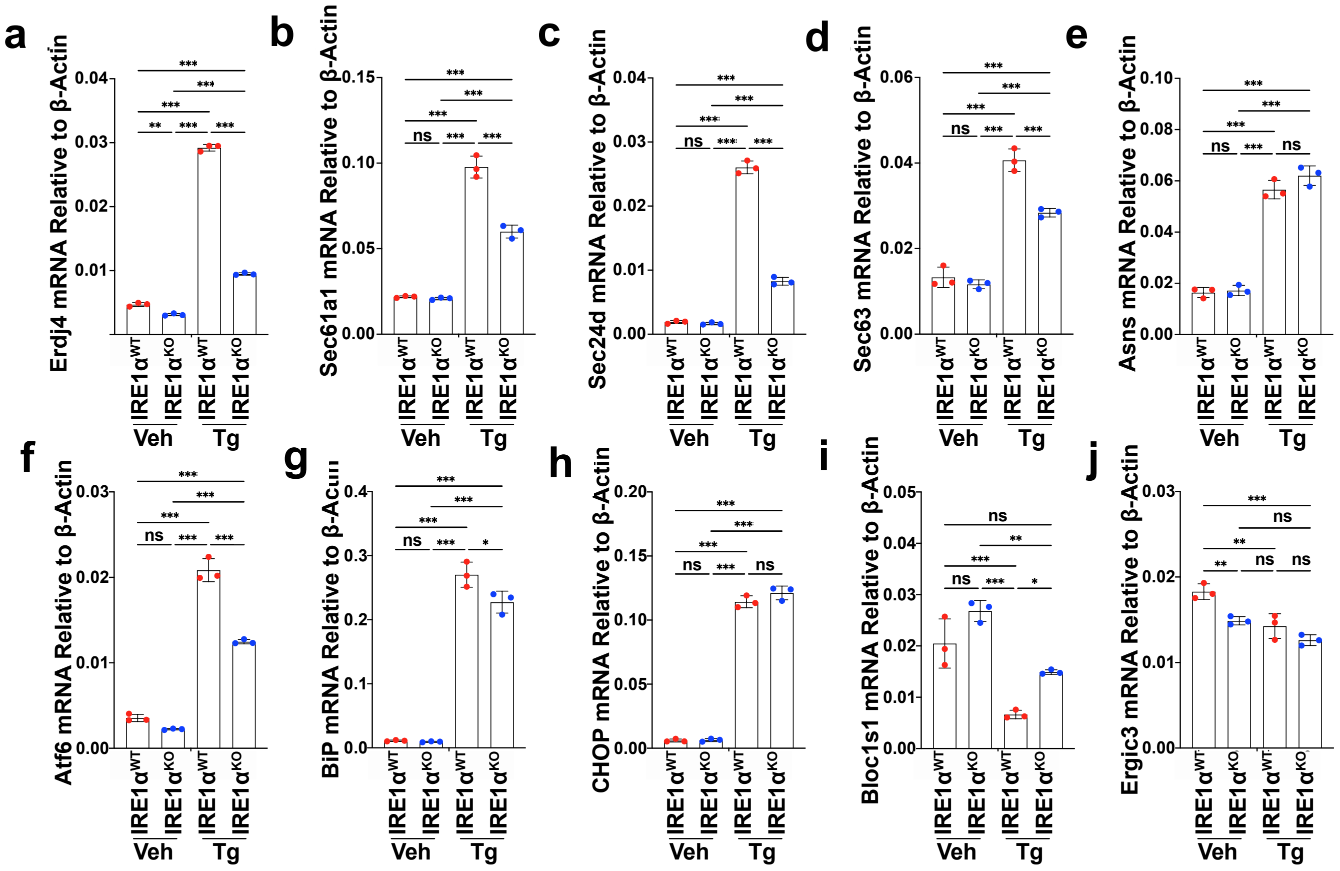

Supplementary Fig. 4. *IRE1α* deletion does not induce compensatory UPR activation in *IRE1α*^{KO} polyclonal HKP1 cells.

a-j, Barplot of RT-PCR of *IRE1α*^{WT} or *IRE1α*^{KO} HKP1 cells (n=3 for each of the four conditions) treated with vehicle or 1μM Tg for 6 hours to evaluate canonical XBP1s target genes Erdj4 (**a,** Veh WT vs Tg WT, p<0.0001, Veh WT vs Veh KO, p=0.0015, Veh WT vs Tg KO, p <0.0001, Tg WT vs Veh KO, P <0.0001, Tg WT vs Tg KO, p <0.0001, Veh KO vs Tg KO, p <0.0001), Sec61a1 (**b,** Veh WT vs Tg WT, p <0.0001, Veh WT vs KO Veh, p=NS, Veh WT vs KO Tg, p <0.0001, Tg WT vs Veh KO, p <0.0001, Tg WT vs Tg KO, p <0.0001, Veh KO vsTg KO p <0.0001), Sec24d (**c,** Veh WT vs Tg WT, p <0.0001, Veh WT vs Veh KO p=NS, Veh WT vs Tg KO, p <0.0001, Tg WT vs Veh KO, p <0.0001 , Tg WT vs Tg KO p <0.0001, VehKO vs Tg KO, p <0.0001), Sec63 (**d,** Veh WT vs Tg WT, p=NS, Veh WT vs VehKO p <0.0001, Veh WT vs TgKO p <0.0001, Tg WT vs Veh KO, p <0.0001, Tg WT vs Tg KO, p <0.0001, Veh KO vs Tg KO, p=0.0002), global UPR markers Asns (**e,** Veh WT vs Tg WT p = NS, Veh WT vs Veh KO, p <0.0001, Veh WT vs Tg KO, p <0.0001, Tg WT vs KO Veh p <0.0001, Tg WT vs TgKO p <0.0001, Veh KO vs TgKO p=NS), Atf6 (**f,** Veh WT vs Tg WT, p = ns, Veh WT vs KO Veh p <0.0001, Veh WT vs KO Tg p <0.0001, Tg WT vs KO Veh p <0.0001, Tg WT vs TgKO, p <0.0001, VehKO vs TgKO. p <0.0001), BiP (**g,** Veh WT vs Tg WT, p=NS, Veh WT vs Veh KO, p <0.0001, Veh WT vs KO Tg p <0.0001, Tg WT vs KO Veh p <0.0001, Tg WT vs KO Tg p <0.0001, KO Veh vs KO Tg p=0.0159), CHOP (**h,** Veh WT vs Tg WT p=NS, Veh WT vs KO Veh p <0.0001, Veh WT vs KO Tg p <0.0001, Tg WT vs KO Veh p <0.0001, Tg WT vs KO Tg p <0.0001, KO Veh vs KO Tg p=NS) and RIDD target genes (Bloc1s1 (**i,** Veh WT vs Tg WT. p=0.072, Veh WT vs VehKO, p=0.0009, Veh WT vs TgKO, p=NS, Tg WT vs KO Veh, p <0.0001, Tg WT vs TgKO p, =0.0025, VehKO vs TgKO p=0.0209), and Ergic3 (**j,** Veh WT vs Tg WT, p=0.0094, Veh WT vs KO Veh p=0.0034, Veh WT vs KO Tg p=0.0003, Tg WT vs VehKO, p=0.8467, Tg WT vs TgKO, p = 0.0703, VehKO vs TgKO, p =NS)**.** 1-way ANOVAs with Tukey's multiple comparisons test; **P* < 0.05, ***P* < 0.001, ****P* < 0.0001. Data are shown as mean ± SD.

Supplementary Fig. 5

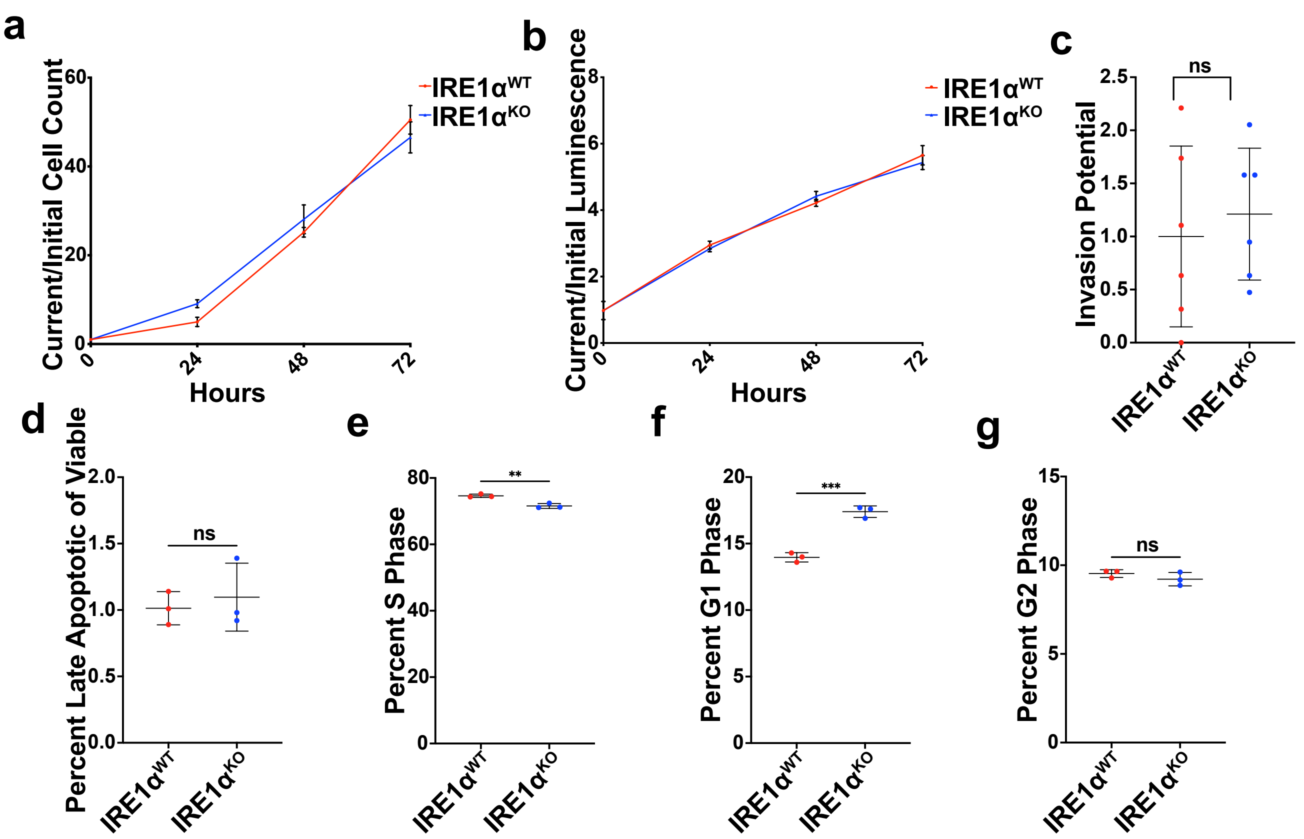

Supplementary Fig. 5. Impact of *IRE1α* loss in *in vitro* cancer cell kinetics in the absence of exogenous ER stress.

Proliferation (**a**), viability (**b**), apoptosis (**c**), invasion potential (**d**), and cell cycle (**e-g**) in *IRE1α*WT (red, n=3 for all assays) or *IRE1α*KO (blue, n=3 for all assays) HKP1 cells. Line graphs are presented with mean values as a symbol ± SD at each time point. Box and whisker plots are presented as mean ± SD. Unless otherwise stated, One-way ANOVA with Tukey's multiple comparisons test for ratios, 2-way ANOVAs with Tukey's multiple comparisons test for all other analyses; *$P < 0.05$, **$P < 0.001$, ***$P < 0.0001$.

Supplementary Fig. 6

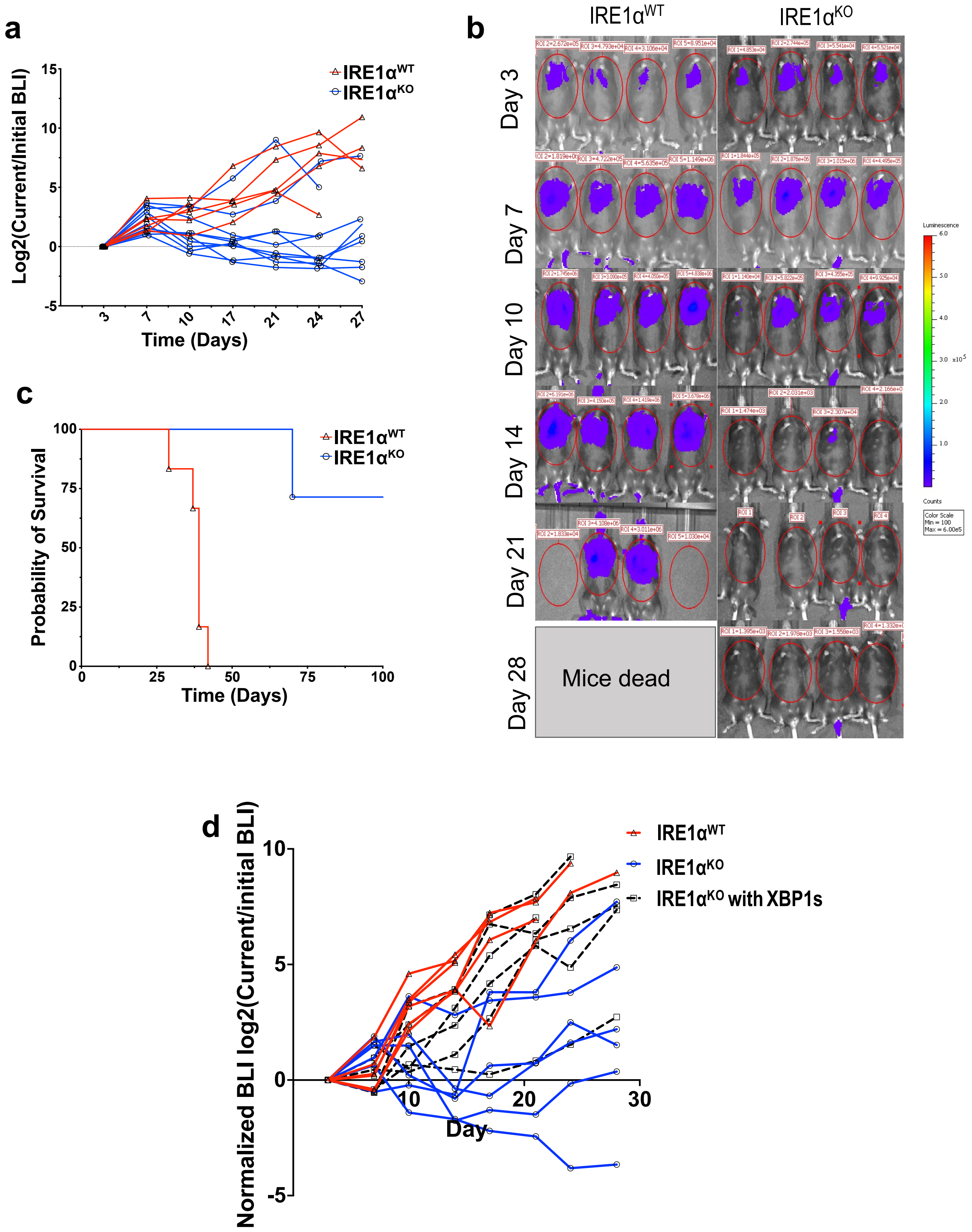

Supplementary Fig. 6. Cancer cell-intrinsic IRE1α deficiency impairs tumor growth and improves survival.

a, BLI spider plots of longitudinally tracked *in vivo IRE1α*^{WT} (red, n = 6) vs *IRE1α*^{KO} (blue, n = 10) HKP1 tumors. Data are shown as individual tracings of mice, relative to their log transformed day 3 measurement. Analyses of different time points in tumor progression were performed using 2-way ANOVA with Tukey's multiple comparisons test; *$P < 0.05$, **$P < 0.001$, ***$P < 0.0001$

b, Biolumuniscence images of representative individual mice as a function of time from IRE1α^{WT} and IRE1α^{KO} mice.

c, Kaplan-Meier plots showing probability of overall survival in *IRE1α*^{WT} (red, n=10) vs *IRE1α*^{KO} (blue, n=10) CMT-167 tumor bearing mice *(*$P < 0.001$*)*.

d, BLI spider plots of longitudinally tracked *in vivo IRE1α*^{WT} empty vector (red, n = 5), *IRE1α*^{KO} empty vector (blue, n = 5) and *IRE1α*^{KO} expressing *Xbp1s* cDNA (black, n = 5) HKP1 tumors. Data are shown as individual tracings of mice, relative to their log transformed day 3 measurement. Data pooled from two independent experiments. *(*$P < 0.001$ for *IRE1α*^{WT} *vs. IRE1α*^{KO} *;* $P<0.002$ *for IRE1α*^{KO} *vs. IRE1α*^{KO} expressing *Xbp1s* cDNA *at day 28).* Analyses of different time points in tumor progression were performed using 2-way ANOVA with Tukey's multiple comparisons test.

Supplementary Fig. 7

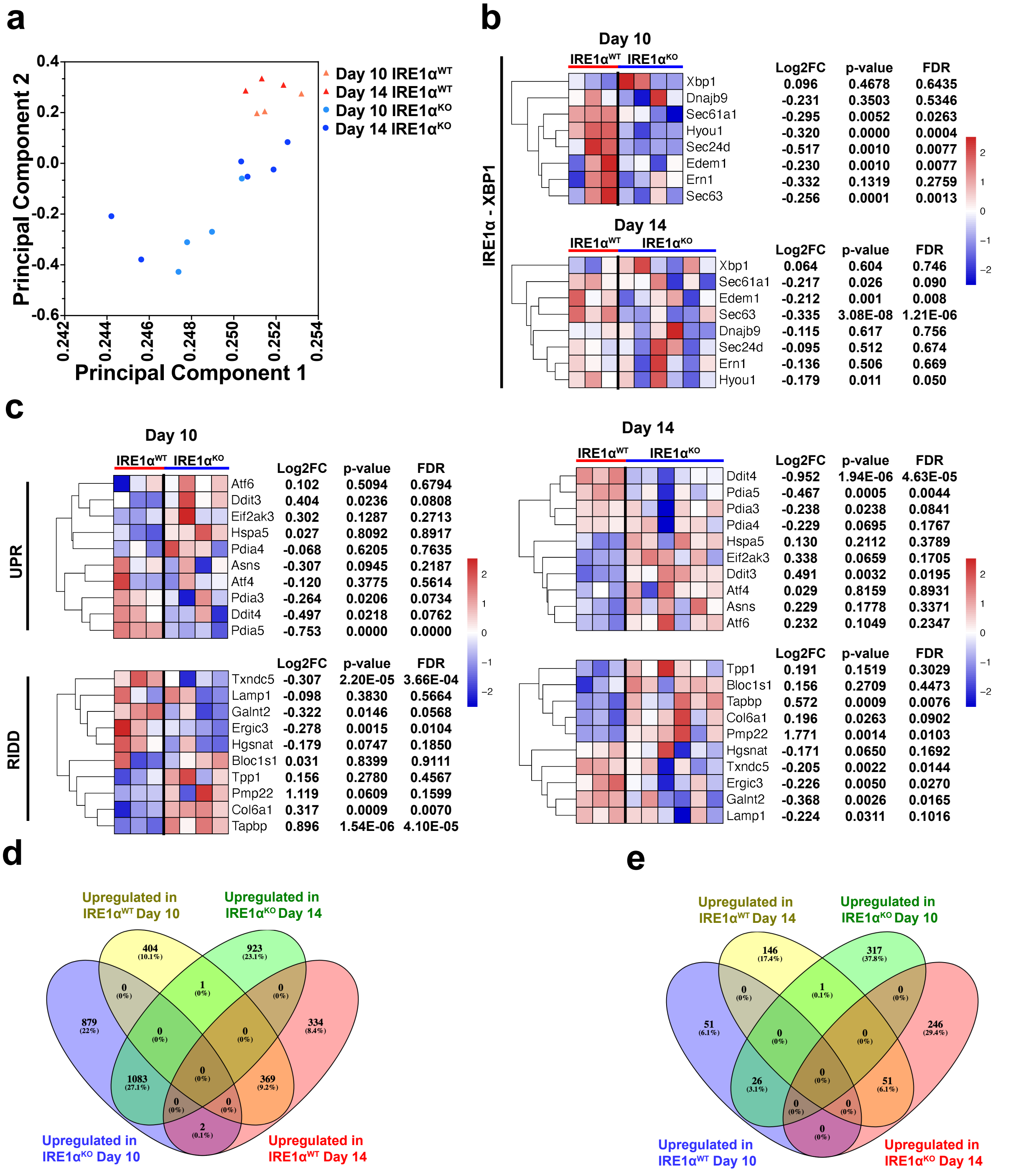

Supplementary Fig. 7. RNA-seq analysis of mcherry+ sorted IRE1α^{WT} vs. IRE1α^{KO} cancer cells from HKP1 tumors.

a, X-Y scatterplot of the first two principal components from mRNA-sequencing of *IRE1α*^{WT} (orange day 10, red day 14) vs *IRE1α*^{KO} (aqua day 10, blue, day 14) HKP1 tumor cells.

b-c, Heatmap of row normalized log2 FPKM values evaluating enrichment for *IRE1α-XBP1*(**a**), UPR and RIDD targets (**b**) in *IRE1α*^{WT} vs *IRE1α*^{KO} HKP1 tumor cells at day 10 and 14.

d-e, Venn diagrams of differentially expressed genes between WT and KO cells over time (**e**) and changes in gene expression within each group over time (**f**). Differentially expressed gene cutoffs were at Log2 Fold Change > 0.5, $P < 0.05$, FDR < 10%.

Supplementary Fig. 8

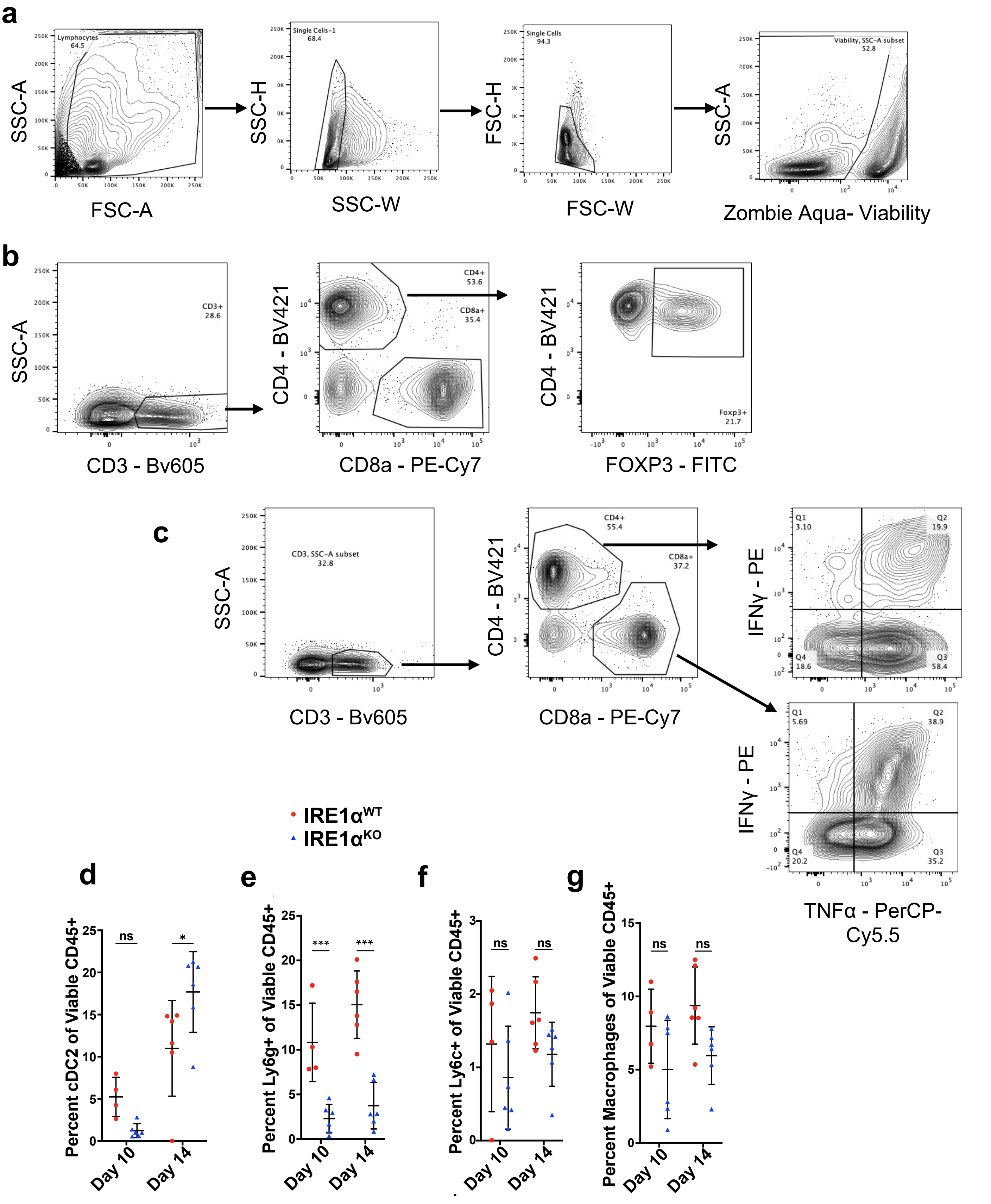

h

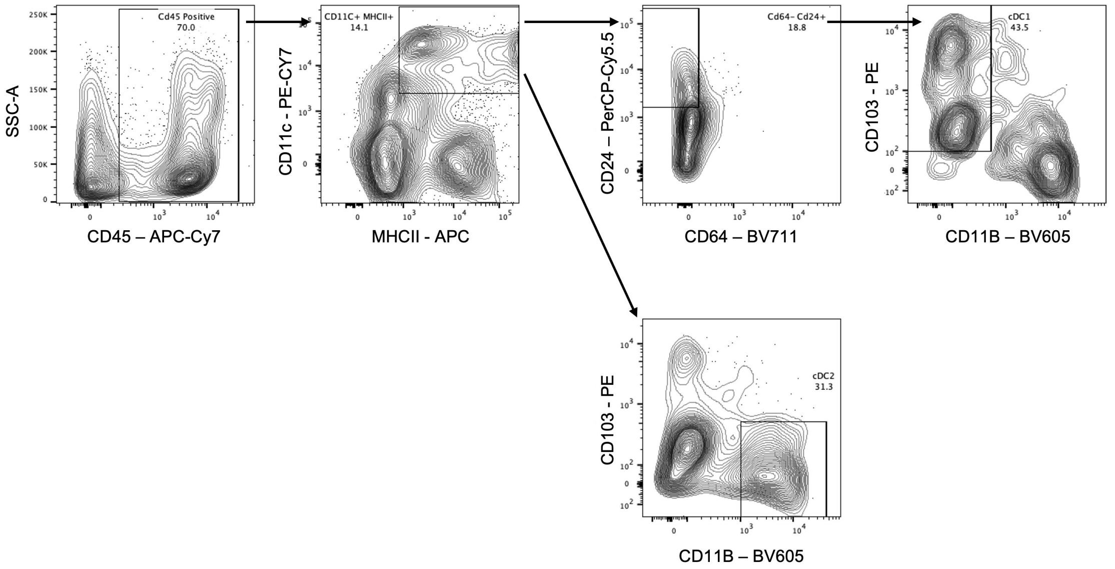

i

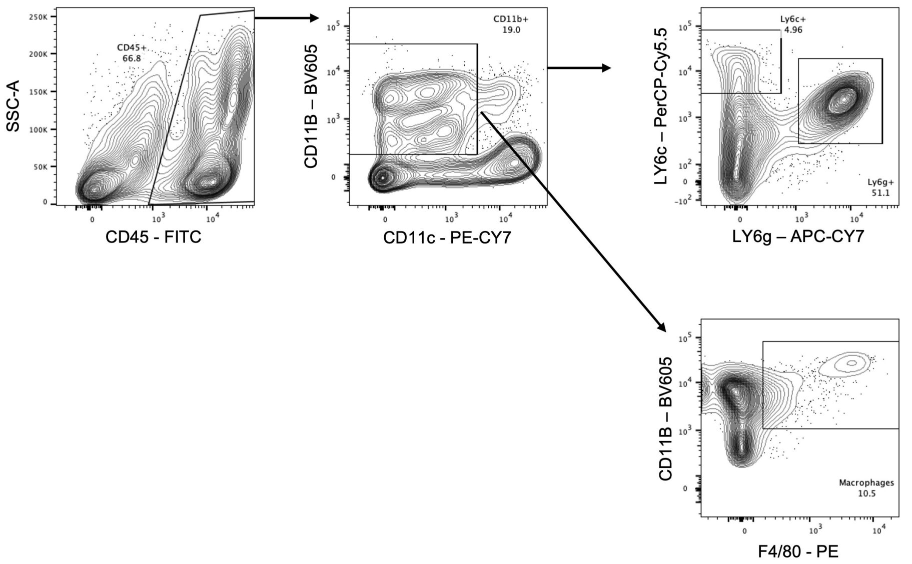

Supplementary Fig. 8. Gating strategy for immune cell populations and quantification of myeloid populations in *IRE1α*^{WT} vs *IRE1α*^{KO} HKP1 tumors.

a-c, Gating strategy for flow cytometry of live/dead cells (**a**), Lymphocytes (**b**), CD4 and CD8 effector T cells (**c**).
d-g, Flow cytometry data from *IRE1α*^{WT} (red) vs *IRE1α*^{KO} (blue) HKP1 tumor bearing lungs at day 10 (n=4 *IRE1α*^{WT} and n=6 *IRE1α*^{KO}) and 14 (n=6 *IRE1α*^{WT} and n=7 *IRE1α*^{KO}), showing conventional dendritic cells type 2 (**d,** day 10, P = NS, day 14, P = 0.0263), PMN-MDSCs (**e,** day 10, P = 0.001, day 14, P<0.0001), Mo-MDSCs (**f,** day 10, P =NS, day 14, P = NS) and Macrophages (**g,** day 10, P= NS, day 14, P = NS). Data are shown as mean ± SD. 2-way ANOVA with Tukey's multiple comparisons test; **P* < 0.05, ***P* < 0.001, ****P* < 0.0001, ns, non-significant.
h-i Gating strategy for flow cytometry of Myeloid cells including cDC1, cDC2 **(h) and** Ly6G and Ly6C MDSCs and Macrophages **(i).**

Unless otherwise stated, One-way ANOVA with Tukey's multiple comparisons test for ratios, 2-way ANOVAs with Tukey's multiple comparisons test for all other analyses; **P* < 0.05, ***P* < 0.001, ****P* < 0.0001.

Supplementary Fig. 9

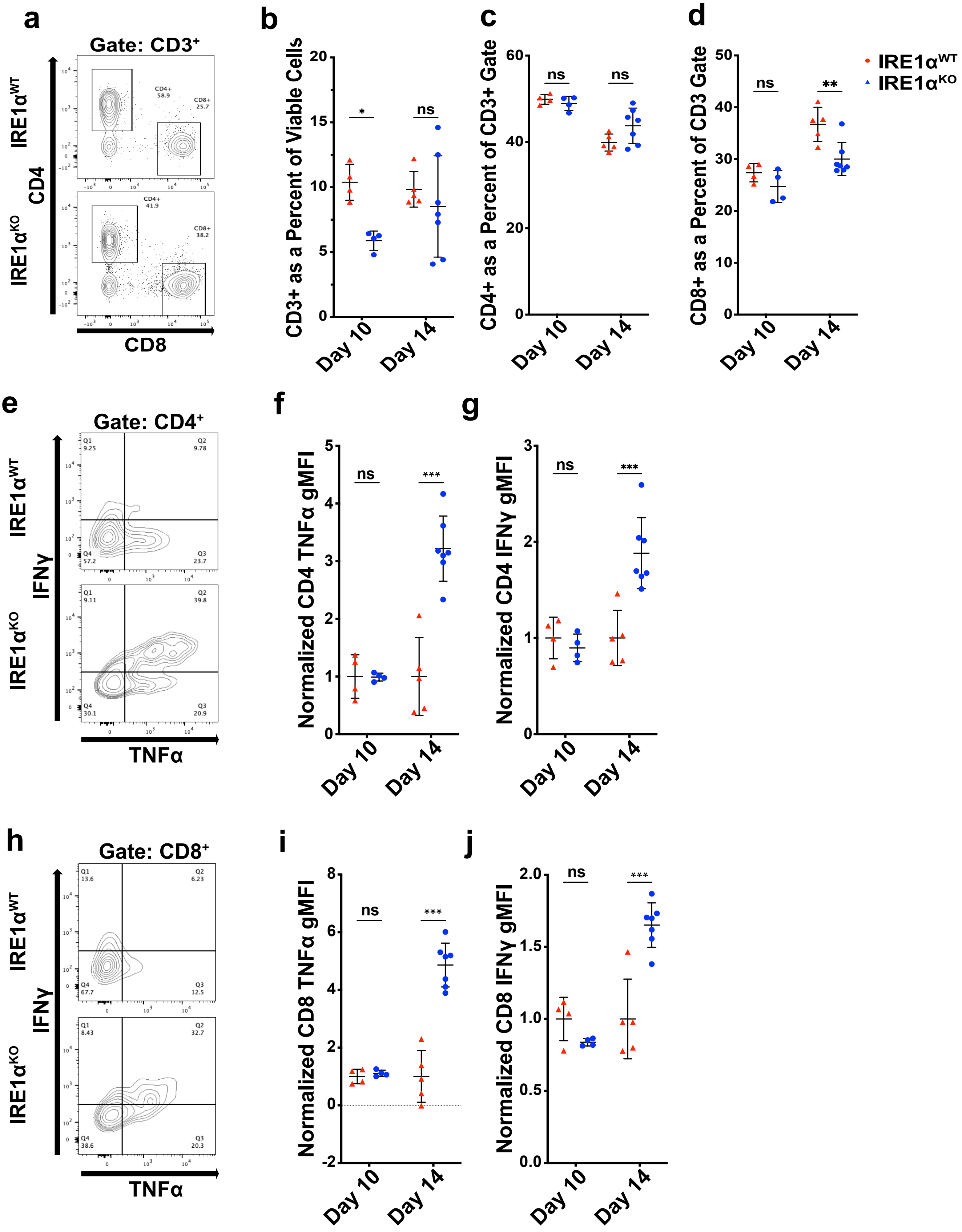

Supplementary Fig. 9. Quantification of T cells in IRE1α^{WT} and IRE1α^{KO} HKP1 tumor bearing lungs at days 10 and 14.

a, Representative flow cytometry scatter plot showing CD4 and CD8 T cells (Gated for CD3) from *IRE1α*^{WT} and IRE1α^{KO} HKP1 lungs.

b-d, Flow cytometry data from *IRE1α*^{WT} (red) vs *IRE1α*^{KO} (blue) HKP1 tumor bearing lungs at day 10 (n=4, *IRE1α*^{WT} and n=6 *IRE1α*^{KO}) and 14 (n=6, *IRE1α*^{WT} and n=7 *IRE1α*^{KO}), of viable CD3 (**b,** day 10 P =0.0113, day 14, P = NS), CD4 (**c,** day 10, P = NS, day 14, P = NS), and CD8 (**d,** day 10, P = NS, day 14, P = 0.003) T lymphocytes. Data are shown as mean ± SD. 2-way ANOVA with Tukey's multiple comparisons test; **P* < 0.05, ***P* < 0.001, ****P* < 0.000, ns, non-significant.

e, Representative flow cytometry scatter plot showing IFNγ/TNFα^{+} T cells (Gated for CD4) from *IRE1α*^{WT} and *IRE1α*^{KO} HKP1 lungs.

f-g, gMFI of normalized TNFα^{+} (**f,** day 10 p =NS, day 14, P = 0.0002) and IFNγ (**g,** day 10, P = NS, day 14, P<0.0001) gMFI for CD4+ T cells from *IRE1α*^{WT} (red) vs *IRE1α*^{KO} (blue) HKP1 tumor bearing lungs at day 10 (n=4, *IRE1α*^{WT} and n=6 *IRE1α*^{KO}) and 14 (n=6, *IRE1α*^{WT} and n=7 *IRE1α*^{KO}). Data are shown as mean ± SD. 2-way ANOVA with Tukey's multiple comparisons test; **P* < 0.05, ***P* < 0.001, ****P* < 0.000, ns, non-significant.

h, Representative flow cytometry scatter plot showing IFNγ/TNFα^{+} T cells (Gated for CD8) from *IRE1α^{WT}* and *IRE1α*^{KO} HKP1 lungs.

i-j, gMFI of normalized TNFα^{+} (**i,** day 10, p = NS, day 14, P<0.0001) and IFNγ (**j,** day 10, P =NS, day 14, P<0.0001) gMFI for CD8+ T cells from *IRE1α*^{WT} (red) vs *IRE1α*^{KO} (blue) HKP1 tumor bearing lungs at day 10 (n=4 *IRE1α*^{WT} and n=6 *IRE1α*^{KO}) and 14 (n=6 *IRE1α*^{WT} and n=7 *IRE1α*^{KO}). Data are shown as mean ± SD. 2-way ANOVA with Tukey's multiple comparisons test; **P* < 0.05, ***P* < 0.001, ****P* < 0.000, ns, non-significant.

Supplementary Fig. 10

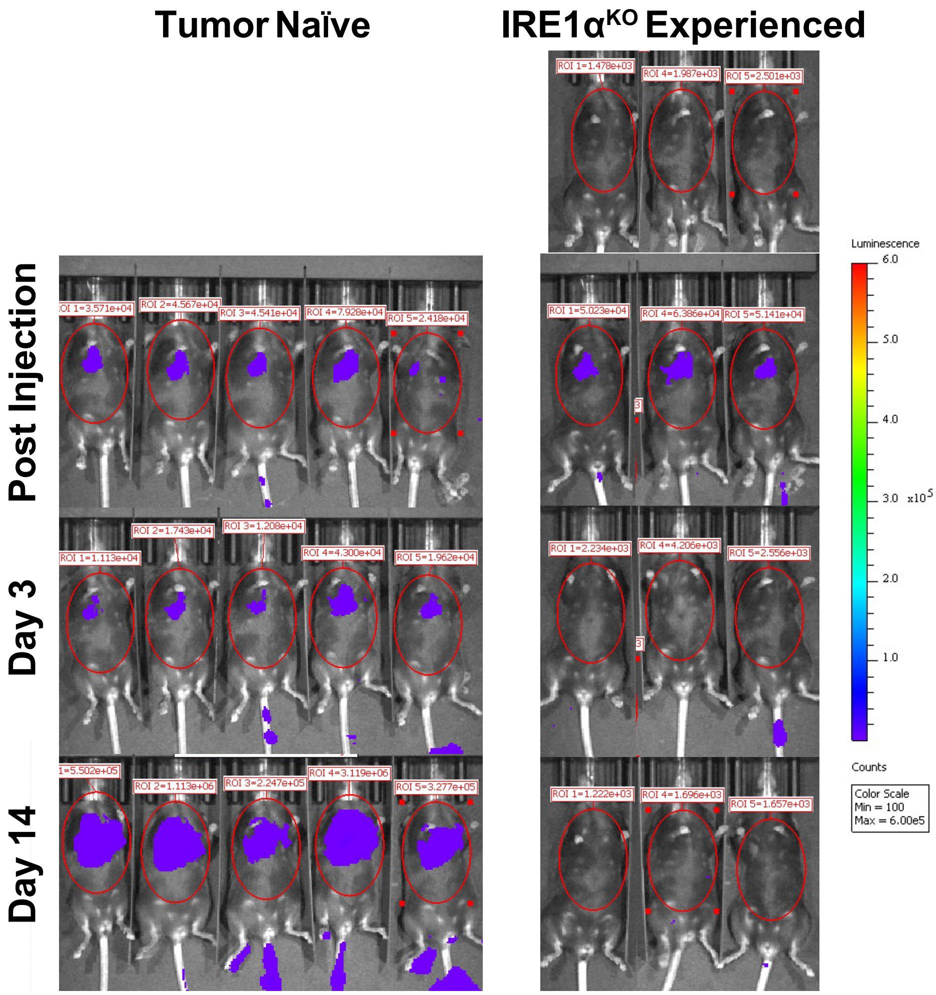

Supplementary Fig. 10: Tumor free mice in the IRE1α^{KO} HKP1 cohort are refractory to tumor re-challenge.

Normalized BLI plots of tumor re-challenge experiments. Aged matched tumor naïve (left) and *IRE1α*^{KO} HKP1 tumor experienced survivor mice (right), prior to, and post administration with parental HKP1.

Unless otherwise stated, One-way ANOVA with Tukey's multiple comparisons test for ratios, 2-way ANOVAs with Tukey's multiple comparisons test for all other analyses; *P < 0.05, **P < 0.001, ***P < 0.0001.

Supplementary Fig. 11

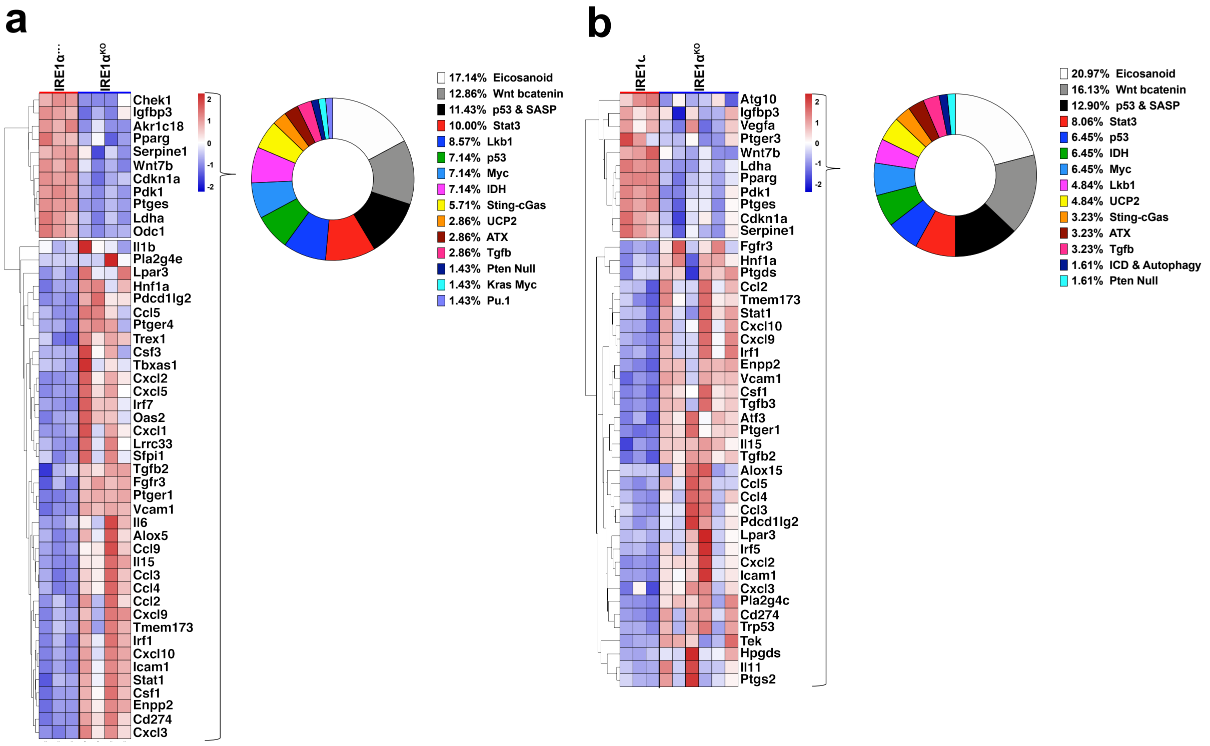

Supplementary Fig. 11.
a-b , Heatmaps of differentially expressed genes between *IRE1α*^{KO} vs. *IRE1α*^{WT} HKP1 cells from the Immunomodulator database at day 10 (**a**), and 14 (**b**).

Supplementary Fig. 12

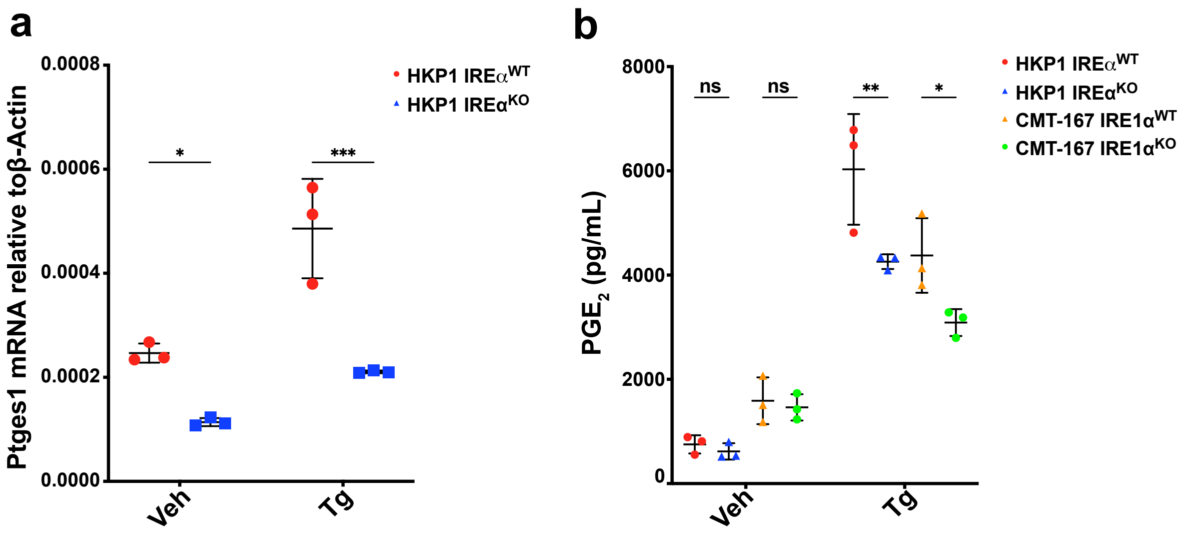

Supplementary Fig. 12. Reduced PGE_2 production in IRE1α^{KO} HKP1 and CMT-167 cells compared to IRE1α^{WT} counterparts

a, qPCR of *Ptges* from 12 hour 1 μM Tg treated IRE1α^{WT} (red) and *IRE1α*^{KO} (blue) HKP1 (n=3, for each of the four conditions). Data are shown as mean ± SD. 2-way ANOVA with Tukey's multiple comparisons test; **P* < 0.05, ***P* < 0.001, ****P* < 0.0001, ns, non-significant.

b, PGE_2 ELISA from 12 hour Vehicle (HKP1, P=NS, CMT-167, P=NS) or Tg (HKP1, P=0.0084, CMT-167, P=0.0178) treated *IRE1α*^{WT} HKP1 (red), *IRE1α*^{KO} HKP1 (blue), *IRE1α*^{WT} CMT-167 (orange), and *IRE1α*^{KO} CMT-167 (green) cells (n=3, for each of the eight conditions). Data are shown as mean ± SD. 2-way ANOVA with Tukey's multiple comparisons test; **P* < 0.05, ***P* < 0.001, ****P* < 0.0001, ns, non-significant.

Data are presented as individual values with box and whisker plots highlighting the median values, and interquartile ranges. Unless otherwise stated, One-way ANOVA with Tukey's multiple comparisons test for ratios; **P* < 0.05, ***P* < 0.001, ****P* < 0.0001.

Supplementary Fig. 13

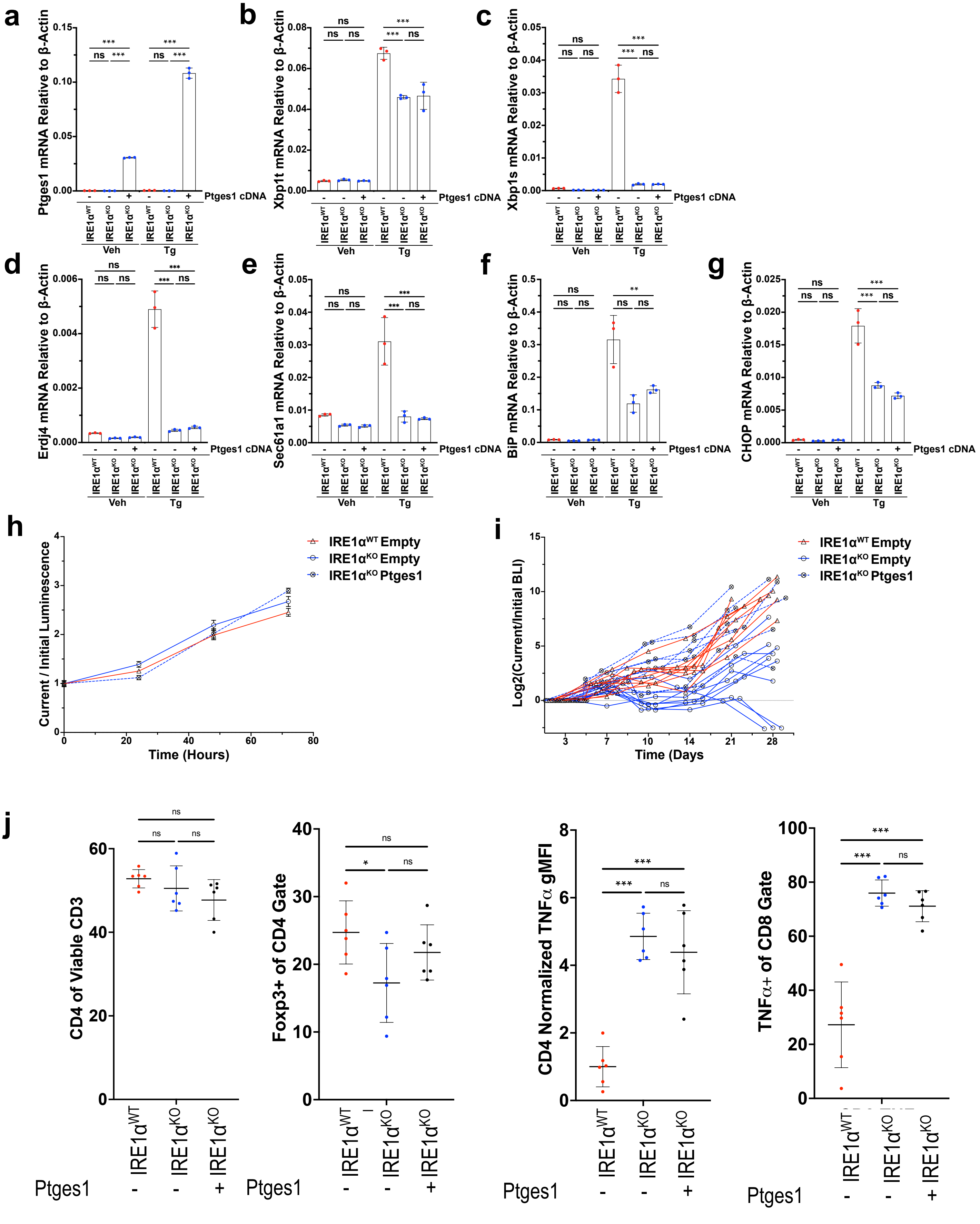

k

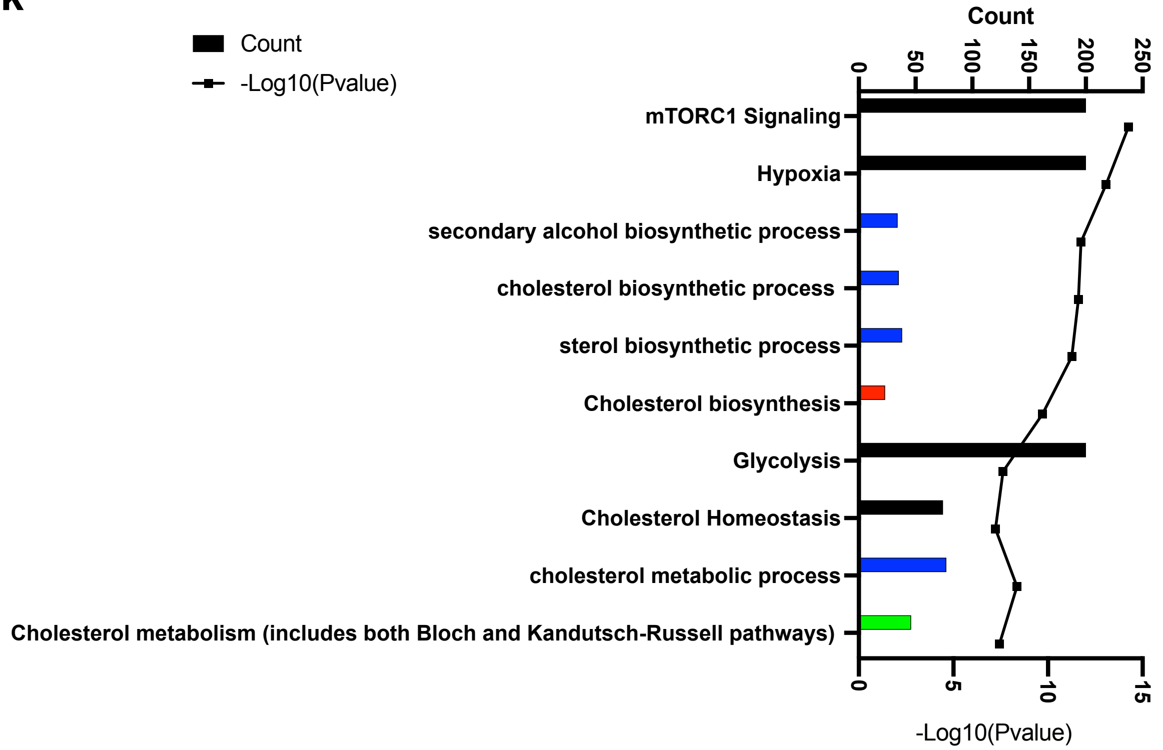

Supplementary Fig. 13. ***Ptges*** **rescue assays in IRE1α^{WT} or IRE1α^{KO} HKP1 cells and evaluation of cell kinetics**.

a-g, Barplots of RT-PCR of Ptges1 (**a**, WT Veh vs KO Veh, p=NS, WT Veh vs KO^{Ptges1} Veh p<0.001, KO Veh vs KO^{Ptges1} Veh, p<0.0001, WT Tg vs KO Tg p=NS, WT Tg vs KO^{Ptges1} Tg p<0.0001, KO Tg vs KO^{Ptges1} Tg p<0.0001), *total Xbp1* (**b**, WT Veh vs KO Veh p=NS, WT Veh vs KO^{Ptges1} Veh p=NS, KO Veh vs KO^{Ptges1} Veh p=NS, WT Tg vs KO Tg p<0.0001, WT Tg vs KO^{Ptges1} Tg p<0.0001, KO Tg vs KO^{Ptges1} Tg p=NS), *Xbp1s* (**c**, WT Veh vs KO Veh p=NS, WT Veh vs KO^{Ptges1} Veh p=NS, KO Veh vs KO^{Ptges1} Veh p=NS, WT Tg vs KO Tg p<0.0001, WT Tg vs KO^{Ptges1} Tg p<0.0001, KO Tg vs KO^{Ptges1} Tg p=NS), XBP1s target genes Erdj4 (**d**, WT Veh vs KO Veh p=NS, WT Veh vs KO^{Ptges1} Veh p=NS, KO Veh vs KO^{Ptges1} Veh p=NS, WT Tg vs KO Tg p<0.0001, WT Tg vs KO^{Ptges1} Tg p<0.0001, KO Tg vs KO^{Ptges1} Tg p=NS), Sec61a1 (**e**, WT Veh vs KO Veh p=NS, WT Veh vs KO^{Ptges1} Veh p=NS, KO Veh vs KO^{Ptges1} Veh p=NS, WT Tg vs KO Tg p<0.0001, WT Tg vs KO^{Ptges1} Tg p<0.0001, KO Tg vs KO^{Ptges1} Tg p=NS), and global UPR genes BiP (**f**, WT Veh vs KO Veh p=NS, WT Veh vs KO^{Ptges1} Veh p=0.0121, KO Veh vs KO^{Ptges1} Veh p=0.0115, WT Tg vs KO Tg p<0.0001, WT Tg vs KO^{Ptges1} Tg p=0.0009, KO Tg vs KO^{Ptges1} Tg p=NS), CHOP (**g**, WT Veh vs KO Veh p=NS, WT Veh vs KO^{Ptges1} Veh, p=NS, KO Veh vs KO^{Ptges1} Veh p=NS, WT Tg vs KO Tg p<0.0001, WT Tg vs KO^{Ptges1} Tg p<0.005, KO Tg vs KO^{Ptges1} Tg, p=NS)) in IRE1α^{WT} empty vector (red) vs IRE1α^{KO} empty vector or *Ptges* cDNA HKP1 cells (blue), treated with vehicle or 6 hours 1 μM Thapsigargin (n=3, for each of the 6 conditions). Data are shown as mean ± SD. 2-way ANOVA with Tukey's multiple comparisons test; *P < 0.05, **P < 0.001, ***P < 0.0001, ns, non-significant.

h, Cell viability in IRE1α^{WT} empty vector (red), *IRE1α*^{KO} empty vector (light blue), and *IRE1α*^{KO} Ptges1 cDNA HKP1 cells (blue), evaluated for 72 hours (WT vs KO p=NS, WT vs KO^{Ptges1} p=NS, KO vs KO^{Ptges1} p=NS). Line graphs are presented with mean values as a symbol ± SD at each time point. Analyses of different time points were performed using 2-way ANOVA with Tukey's multiple comparisons test; *P < 0.05, **P < 0.001, ***P < 0.0001.

i, BLI spider plots of longitudinally tracked *in vivo* *IRE1α*^{WT} empty vector (red, n = 10), *IRE1α*^{KO} empty vector (blue, n = 10) and *IRE1α*^{KO} *Ptges* cDNA (blue broken line, n = 10) HKP1 tumors. Data are shown as individual tracings of mice, relative to their log transformed day 3 measurement. Analyses of different time points in tumor progression were performed using 2-way ANOVA with Tukey's multiple comparisons test; $*P < 0.05$, $**P < 0.001$, $***P < 0.0001$

j. Flow cytometry data for CD4 (WT vs KO, p=NS, WT vs KO^{Ptges1},P=NS, KO vs KO^{Ptges1}, P=NS), T-regs (WT vs KO P=0.0467, WT vs KO^{Ptges1} P=NS, KO vs KO^{Ptges1} P=NS), CD4 normalized TNFα^{+} (WT vs KO, P<0.0001, WT vs KO^{Ptges1} P<0.0001, KO vs KO^{Ptges1}, P=NS) and CD8 normalized TNFα^{+} (WT vs KO, P<0.0001, WT vs KO^{Ptges1} ,P<0.0001, KO vs KO^{Ptges1} p=NS) from *IRE1α*^{WT} (red, n=6) vs *IRE1α*^{KO} (blue, n=6) and *IRE1α*^{KO} with Ptges1 (black, n=6) HKP1 tumor bearing lungs (Box and whisker plots are presented as mean ± SD.

k. Pathway enrichments in *IRE1α*^{KO} downregulated signature genes. Count of genes is represented as a barplot, and the plotted –log10p-values are represented as a dot above its corresponding bar.

Supplementary Fig. 14

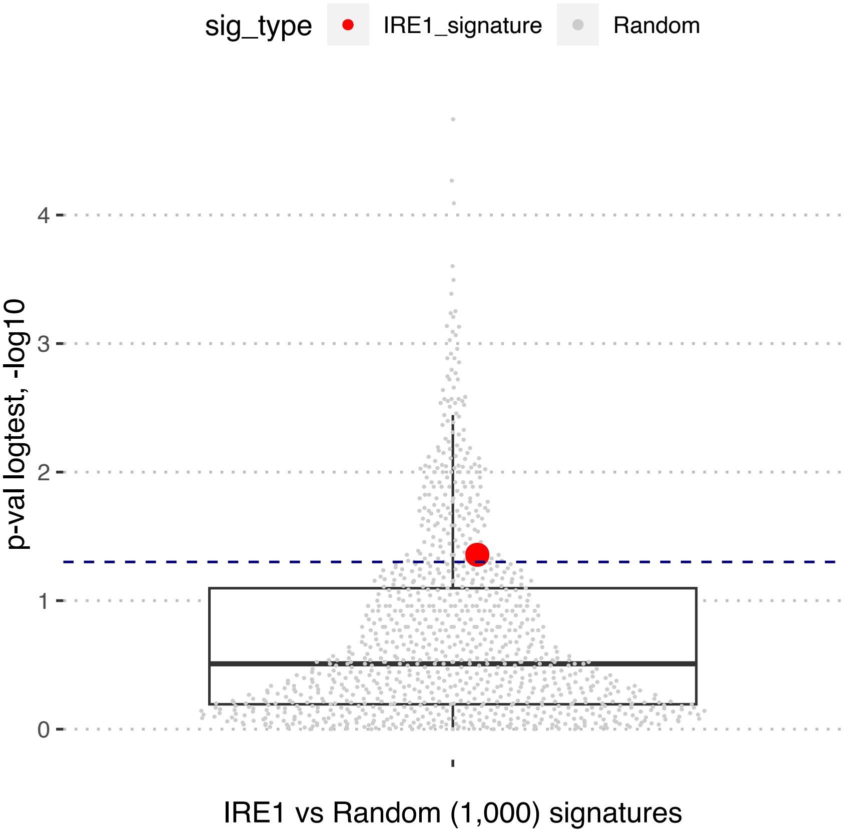

Supplementary Fig. 14. Multivariate analysis showing OS) and *IRE1α*^{KO} signature status.

Multivariate Cox proportional-hazards regression models for IRE1α versus 1,000 random signatures of identical lengths. Red dot denotes the p-values from survival model for IRE1α signature and gray dots denote the p-values from survival model for random signatures. Blue dashed line denotes p-values of 0.05. Out of n=1,001 signatures (1 IRE1α and 1,000 random), 178/1000 random signatures were significant in multivariate survival models. So, specificity = 100 * TN/(FP + TN) = 100* (822 / (178+822)) = 82.2%. Data are presented as a box and whisker plot, with the median value highlighted, and interquartile ranges.

Supplementary Fig. 15

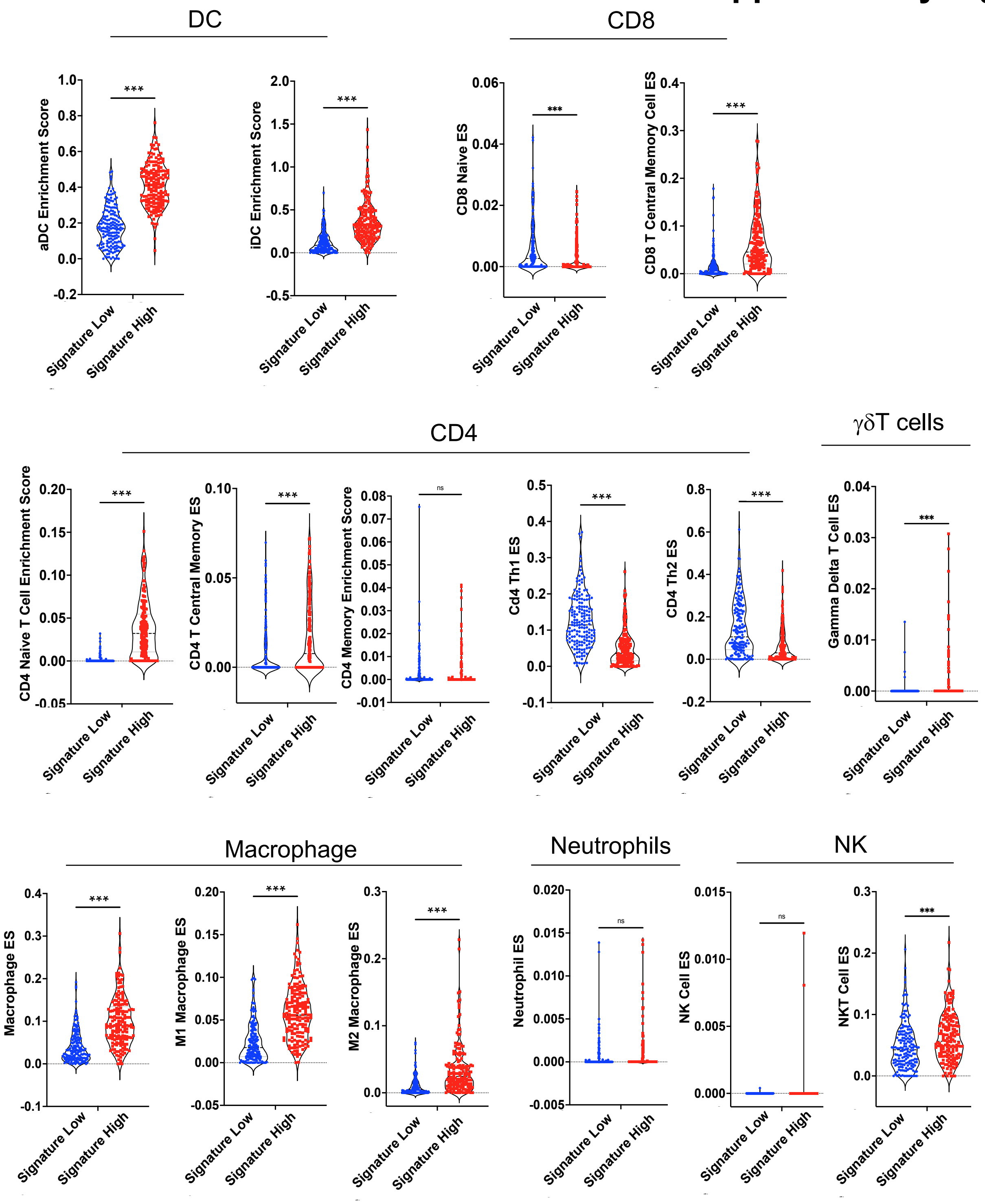

Supplementary Fig. 15. Signature evaluation, RNA-seq analysis and deconvolution.

Violin plots of xCell pipeline deconvolution of TCGA LUAD patients from the high (red, n=166) or low (blue, n=165) groups showing enrichment of aDC (P<0.0001), iDC (P<0.0001), CD8 naïve (P=0.0001), CD8 central memory (P<0.0001), CD4 naïve (P<0.0001), CD4 TCM (P<0.0001), CD4 memory(p=NS), CD4 Th1 (p<0.0001), Cd4 Th2 (P<0.0001), Gamma delta (P<0.0001), Macrophages (p<0.0001), M1 macrophages (P<0.0001), M2 macrophages (P<0.0001), Neutrophil (p=NS), NK (p=NS), and NKT (P=0.0005). Unpaired Student's t-test. **P* < 0.05, ***P* < 0.001, ****P* < 0.0001.

Supplementary Table 1.

	N=232		
	XBP1s Low N=116	*XBP1s* High N=116	'p' value
**Gender**			
Female	61 (53%)	66 (57%)	p=0.510
Male	55 (47%)	50 (43%)	
**Smoking**			
No (1 or 2)	51 (45%)	44 (39%)	p=0.344
Yes (2 or 3)	61 (55%)	68 (61%)	
**Path stage**			
IA/IB	62 (53%)	57 (49%)	p=0.796
IIA/IIB	31 (27%)	33 (28%)	
III/IV	23 (20%)	26 (22%)	
**Path 'T' stage**			
T1a/T1b	30 (26%)	37 (32%)	
T2a/T2b	72 (62%)	64 (55%)	p=0.539
T3/T4	14 (12%)	15 (13%)	
**Path 'N' status**			
Nx/N0	76 (66%)	71 (61%)	p=0.496
Positive LN (N1/N2]	40 (34%)	45 (39%)	
**Neo-adjuvant**			
No	116 (100%)	114 (99%)	p=0.314
Yes	0 (0%)	1 (1%)	
**Vital status**			
Death	26 (22%)	36 (31%)	p=0.138

Supplementary Table 1: *XBP1s* enrichment does not correlate with clinicopathologic factors.
Evaluation of gender, smoking, pathologic stage, T, N and M stages, neoadjuvant treatments with *XBP1s* High (n=116) or Low (n=116) status. All comparisons performed using the chi-square test. * denotes $P < 0.05$, ** denotes $P < 0.001$, *** denotes $P < 0.0001$

Supplementary Table 2.

Characteristic	HR[1,2]	SE[2]	95% CI[2]	p-value
XBP1_Splicing_Status				
High	—	—	—	
Low	0.60*	0.241	0.38, 0.97	0.037
Diagnosis.Age	1.01	0.013	0.99, 1.04	0.3
Path_stage				
Stage I	—	—	—	
Stage II	0.86	0.530	0.30, 2.43	0.8
Stage III	0.97	0.636	0.28, 3.37	>0.9
Stage IV	0.92	0.741	0.22, 3.95	>0.9
Path_T_stage				
T1	—	—	—	
T2	1.17	0.288	0.66, 2.05	0.6
T3/T4	2.86*	0.485	1.11, 7.39	0.030
Path_N_stage				
Nx/N0	—	—	—	
N1/N2/N3	2.56	0.505	0.95, 6.89	0.063
Smoking_history				
Non-Smoker	—	—	—	
Current Smoker	1.27	0.412	0.57, 2.84	0.6
Current Smoker Reformed	1.55	0.376	0.74, 3.24	0.2

[1] *p<0.05; **p<0.01; ***p<0.001

[2] HR = Hazard Ratio, SE = Standard Error, CI = Confidence Interval

Supplementary Table 2. Multivariate analysis showing overall survival (OS) and *XBP1s* in TCGA-LUAD. High is upper tertile and low is bottom tertile of the XBP1 Splicing Scores. Low XBP1 group is reference population. HR is hazard ratio and p is log-rank p-value from the multivariate Cox proportional-hazards regression model adjusted for age at the time of diagnosis, gender, pathology and TN stages.

Supplementary Table 3

Log2 FC	FDR	P-value	Up/down	# Genes	25% OS	33% OS
-	0.01	0.05	Up	1054	0.0026	0.016
-	0.01	0.05	Down	1179	0.00068	<0.0001
1.00	0.01	0.05	Up	582	0.0054	0.0043
1.00	0.01	0.05	Down	144	0.032	0.03
1.00	0.001	0.05	Up	323	0.013	0.019
1.00	0.001	0.05	Down	95	0.068	0.07
1.50	0.001	0.05	Up	238	0.00087	0.0065
1.50	0.001	0.05	Down	38	0.44	0.61
2.00	0.001	0.05	Up	171	0.00045	0.0041
2.00	0.001	0.05	Down	21	0.81	0.99

Supplementary Table 3. IRE1α^{KO} gene signatures with indicated cut-off values. Log2FC>1 and FDR 0.01 highlighted in red.

Supplementary Table 4

Target Gene	Species	Forward Sequence	Reverse Sequence	Experiment
Xbp1t	Mouse	GACAGAGAGTCAAACTAACGTGG	GTCCAGCAGGCAAGAAGGT	qPCR
Xbp1s	Mouse	AAGAACACGCTTGGGAATGG	CTGCACCTGCTGCGGAC	qPCR
Erdj4	Mouse	TAAAAGCCCTGATGCTGAAGC	TCCGACTATTGGCATCCGA	qPCR
Sec61a1	Mouse	CTATTTCCAGGGCTTCCGAGT	AGGTGTTGTACTGGCCTCGGT	qPCR
Sec24d	Mouse	TCCACTCTCCCCATGGTTTA	GCTATATCCGCTGCACTACG	qPCR
Sec63	Mouse	GTGGACTACAGCGTTTGCACA	CATATCAGCCCTCACTGCTGC	qPCR
Edem1	Mouse	AAGCCCTCTGGAACTTGCG	AACCCAATGGCCTGTCTGG	qPCR
Hyou1	Mouse	TGCGCTTCCAGATCAGTCC	GGAGTAGTTCAGAACCATGCC	qPCR
BiP	Mouse	TCATCGGACGCACTTGGAA	CAACCACCTTGAATGGCAAGA	qPCR
Chop	Mouse	GTCCCTAGCTTGGCTGACAGA	TGGAGAGCGAGGGCTTTG	qPCR
Atf6	Mouse	GACTCACCCATCCGAGTTGTG	CTCCCAGTCTTCATCTGGTCC	qPCR
Ptges	Mouse	AGCACACTGCTGGTCATCAA	TTGGCAAAAGCCTTCTTCCGC	qPCR
Bloc1s1	Mouse	CACCCAGCCAGACTCGAC	GCAGCGATAGCTTCTCTCCTC	qPCR
Ergic3	Mouse	GAAGCAGTTCGATGCCTACCC	ACTCCGATAGGAAAAGCAGGA	qPCR
Gapdh	Mouse	GGTCCTCAGTGTAGCCCAAG	AATGTGTCCGTCGTGGATCT	qPCR
b-actin	Mouse	CTAAGGCCAACCGTGAAAAG	ACCAGAGGCATACAGGGACA	qPCR
IRE1α_crRNA	Mouse	TCAGATGGAATCCTCTACAT	NA	NEON Cas9 Editing
IRE1α Sequencing	Mouse	AGAGTGACAACAGCCTGCTC	CCAGCAAGAGACCCCATCTG	Sequencing
Mouse Ptges1 Cloning	Mouse	ATGAATTCGCCACCATGCCTTCCCCGGG CCTGGTGATG	ATGCGGCCGCTCACAGATGGTGG GCCACCTCCC	Rescue Cloning
Mouse Xbp1s Cloning	Mouse	ATGAATTCGCCACCATGGTGGTGGTGG CAGCGGCGCC	ATGCGGCCGCTTAGACACTAATCA GCTGGGGG	Rescue Cloning

Supplementary Table 4. qPCR, CRISPR-Cas9 editing sanger sequencing, and cloning primers

Supplementary Table 5

Target Gene	Clone	Fluorophore	Company	Dilution	Experim ent
CD103	2E7	PE	BioLegend	1 to 100	FACS
CD11b	M1/70	BV605	BioLegend	1 to 100	FACS
Zombie-Aqua	NA	Bv510	Biolegend	1 to 100	FACS
MHC I (H-2Kb/H-2Db)	28-8-6	FITC	BioLegend	1 to 100	FACS
CD45	30-F11	APC-Cy7	BioLegend	1 to 100	FACS
CD11c	N418	PE-Cy7	BioLegend	1 to 100	FACS
CD24	30-F1	Percp-Cy5.5	BioLegend	1 to 100	FACS
Ly6g	1A8	Bv421 / Pac Blue/ Dapi	BioLegend	1 to 100	FACS
CD64	10.1	Bv711	BioLegend	1 to 100	FACS
Ly6c	Hk1.4	Bv785	Biolegend	1 to 100	FACS
IFNg	XMG1.2	FITC	Biolegend	1 to 100	FACS
TNFa	mAb11	Percp-Cy5.5	Biolegend	1 to 100	FACS
F4/80	QA17A29	Pacific Blue	Biolegend	1 to 100	FACS
CD3	17A2	PE	BioLegend	1 to 100	FACS
CD8a	53-6.7	PE-Cy7	BioLegend	1 to 100	FACS
Gzmb	QA16A02	APC / A-647	BioLegend	1 to 100	FACS
Cd4	RM4-5	Pacific Blue	BioLegend	1 to 100	FACS
Foxp3	FJK-16s	FITC	BioLegend	1 to 100	FACS
Ki-67	16A8	APC / A-647	BioLegend	1 to 100	FACS
PD1 (Cd279)	29F.1A12	APC-Cy7	BioLegend	1 to 100	FACS
XBP1s	Q3-695	AF647	BD Biosciences	1 to 100	FACS
IRE1α	14C10	NA	Cell Signaling	1 to 1000	WB
XBP1s	9D11A43	NA	Biolegend	1 to 1000	WB

Supplementary Table 5 Flow Cytometry and western blot antibodies
