# Supplementary material for: Tumor-intrinsic IRE1α signaling controls protective immunity in lung cancer
Source: Nat Commun. 2023 Jan 9;14:120. doi: 10.1038/s41467-022-35584-9 (PMC9829901; doi:10.1038/s41467-022-35584-9)
Supplement: Supplementary file 2 — Description of Additional Supplementary Files [file 41467_2022_35584_MOESM2_ESM.pdf]

Description of Additional Supplementary Files

Supplementary Data 1: Differentially expressed genes between IRE1αKO vs IRE1αWT HKP1

Supplementary Data 2: Enricher analysis IRE1aKO vs WT (WT all genes)

Supplementary Data 3: Immunomodulator database
