# Supplementary material for: Tumor-intrinsic IRE1α signaling controls protective immunity in lung cancer
Source: Nat Commun. 2023 Jan 9;14:120. doi: 10.1038/s41467-022-35584-9 (PMC9829901; doi:10.1038/s41467-022-35584-9)
Supplement: Supplementary file 6 — Reporting Summary [file 41467_2022_35584_MOESM6_ESM.pdf]

nature portfolio

Corresponding author(s): Juan Cubillos-Ruiz and Vivek Mittal

Last updated by author(s): Nov 14, 2022

Reporting Summary

Nature Portfolio wishes to improve the reproducibility of the work that we publish. This form provides structure for consistency and transparency in reporting. For further information on Nature Portfolio policies, see our Editorial Policies and the Editorial Policy Checklist.

Statistics

For all statistical analyses, confirm that the following items are present in the figure legend, table legend, main text, or Methods section.

n/a	Confirmed	
☐	☒	The exact sample size (n) for each experimental group/condition, given as a discrete number and unit of measurement
☐	☒	A statement on whether measurements were taken from distinct samples or whether the same sample was measured repeatedly
☐	☒	The statistical test(s) used AND whether they are one- or two-sided<br>*Only common tests should be described solely by name; describe more complex techniques in the Methods section.*
☐	☒	A description of all covariates tested
☐	☒	A description of any assumptions or corrections, such as tests of normality and adjustment for multiple comparisons
☐	☒	A full description of the statistical parameters including central tendency (e.g. means) or other basic estimates (e.g. regression coefficient) AND variation (e.g. standard deviation) or associated estimates of uncertainty (e.g. confidence intervals)
☐	☒	For null hypothesis testing, the test statistic (e.g. F, t, r) with confidence intervals, effect sizes, degrees of freedom and P value noted<br>*Give P values as exact values whenever suitable.*
☒	☐	For Bayesian analysis, information on the choice of priors and Markov chain Monte Carlo settings
☐	☒	For hierarchical and complex designs, identification of the appropriate level for tests and full reporting of outcomes
☒	☐	Estimates of effect sizes (e.g. Cohen's d, Pearson's r), indicating how they were calculated

Our web collection on statistics for biologists contains articles on many of the points above.

Software and code

Policy information about availability of computer code

Data collection	NCI Genomics Database Commons (GDC)<br>The cancer genome atlas (TCGA)

Data analysis	SAMtools mpileup (version 1.2)<br>SAMtools (version 1.14)<br>bowtie2 (version 2.3.0)<br>Cufflinks (version 2.2.1)<br>GSEA (version 4.2.2)<br>DNASTAR Lasergene 17 SeqMan Pro<br>FlowJO (version 10.6.2)<br>Pheatmap (version 1.0.12)<br>RColorBrewer (version 1.1-2)<br>Venny (version 2.1)<br>DESEQ2 (version 1.28.1)<br>R (version 4.0.1)<br>R Studio (version 1.3.959)<br>Graphpad prism (version 9.2.0)<br>ggplot2 (version 3.3.3)<br>GSVA (version 1.36.3)<br>Tophat2 (version 2.0.1)<br>https://github.com/akv3001/RNASeq-Based-XBP1s-Detection-.git

For manuscripts utilizing custom algorithms or software that are central to the research but not yet described in published literature, software must be made available to editors and reviewers. We strongly encourage code deposition in a community repository (e.g. GitHub). See the Nature Portfolio guidelines for submitting code & software for further information.

Data

Policy information about availability of data

All manuscripts must include a data availability statement. This statement should provide the following information, where applicable:

- Accession codes, unique identifiers, or web links for publicly available datasets
- A description of any restrictions on data availability
- For clinical datasets or third party data, please ensure that the statement adheres to our policy

The authors declare that the data supporting the findings of this study are available in the manuscript [and its Supplementary Information files]. Source data are provided with this paper.
The publicly available LUAD data used in this study are collected from TCGA (https://portal.gdc.cancer.gov/projects/TCGA-LUAD).
Raw RNA-seq data have been uploaded in the Gene Expression Omnibus database under accession GSE202939 (https://www.ncbi.nlm.nih.gov/geo/query/acc.cgi?acc=GSE202939).
Other datasets generated in the study includes the Immunomodulator database (Supplementary information). The murine IRE1a gene signature was applied to an independent collection of 44 human lung tumors previously reported [REF 31]. Data can be obtained from corresponding author: (https://www.ncbi.nlm.nih.gov/projects/gap/cgi-bin/study.cgi?study_id=phs002818.v1.p1). Human sample data, were downloaded from the NCI Genomics Database Commons (GDC) and The cancer genome atlas (TCGA).

Field-specific reporting

Please select the one below that is the best fit for your research. If you are not sure, read the appropriate sections before making your selection.

☒ Life sciences ☐ Behavioural & social sciences ☐ Ecological, evolutionary & environmental sciences

For a reference copy of the document with all sections, see nature.com/documents/nr-reporting-summary-flat.pdf

Life sciences study design

All studies must disclose on these points even when the disclosure is negative.

Sample size	For in vitro experiments, we used negative and positive controls. n numbers of each group were indicated as for biologic replicates in the figure legends. For in vivo experiments, each cohort comprises n>=5 mice per treatment group. Based on prior mouse experiments, this number will provide general power to detect differences greater than 50% at a significance of p<0.05 (Ban et al. doi: 10.1038/s43018-021-00245-1).
Data exclusions	Any outlier, if at all, was excluded after analyzing the datasets with ROUT method. For example in the in vivo Xbp1s rescue experiment.
Replication	Experiments were repeated, and verification of experiment findings were successful.
Randomization	Tumor burden of mice were quantified by BLI imaging before any given treatment. Mice were then distributed into treatment groups by ranking them by tumor burden and performing an S distribution, ensuring comparable tumor burdens between cohorts.

Blinding	IRE1a gene signature and deconvolution analysis on TCGA and validation cohort was performed blinded. Investigators were not blinded during in vivo or in vitro experiments because the investigator who participated in experimental design, also performed the experiment. IRE1a gene signature and deconvolution analysis on TCGA and validation cohort was blinded.

Reporting for specific materials, systems and methods

We require information from authors about some types of materials, experimental systems and methods used in many studies. Here, indicate whether each material, system or method listed is relevant to your study. If you are not sure if a list item applies to your research, read the appropriate section before selecting a response.

Materials & experimental systems

n/a	Involved in the study
☐	☒ Antibodies
☐	☒ Eukaryotic cell lines
☒	☐ Palaeontology and archaeology
☐	☒ Animals and other organisms
☒	☐ Human research participants
☒	☐ Clinical data
☒	☐ Dual use research of concern

Methods

n/a	Involved in the study
☒	☐ ChIP-seq
☐	☒ Flow cytometry
☒	☐ MRI-based neuroimaging

Antibodies

Antibodies used	All antibodies used in the study are described in the manuscript including the company name, clone number and dilution.
Validation	All primary antibodies used in the study were verified for species specificity as described in the manufacture's website.

Eukaryotic cell lines

Policy information about cell lines

Cell line source(s)	HKP1 lung cancer cells were developed from KrasG12D/p53-/- in our lab (Choi, H et al. Cell Rep. 2015. PMID:25704820). HEK-293 cell line was purchased from ATCC. CMT-167 cell line was purchased from Sigma-Aldrich.
Authentication	Authentication of the cell line was confirmed by genomic analysis.
Mycoplasma contamination	Cells used in the study were mycoplasma contamination free as confirmed routinely with MycoAlert PLUS kit (Lonza)
Commonly misidentified lines (See ICLAC register)	No misidentified lines were used in the study.

Animals and other organisms

Policy information about studies involving animals; ARRIVE guidelines recommended for reporting animal research

Laboratory animals	Wild type C57BL/6J (Stock # 000664) or RAG2 deficient (stock # 008449) mice purchased from the Jackson Laboratory (Bar Harbor, Maine).
Wild animals	No wild animals were involved in the study.
Field-collected samples	The study did not involve samples collected from the field.
Ethics oversight	All animal related works were conducted following protocols approved by the Institutional Animal Care and Use Committee at Weill Cornell Medicine.

Note that full information on the approval of the study protocol must also be provided in the manuscript.

Flow Cytometry

Plots

Confirm that:

☒ The axis labels state the marker and fluorochrome used (e.g. CD4-FITC).

☒ The axis scales are clearly visible. Include numbers along axes only for bottom left plot of group (a 'group' is an analysis of identical markers).

☒ All plots are contour plots with outliers or pseudocolor plots.

☒ A numerical value for number of cells or percentage (with statistics) is provided.

Methodology

Sample preparation	Cells were then stained following the standard flow cytometry protocol. In brief, cells were first stained with Zombie Aqua Fixable Viability dye in accordance with the manufacturers protocol. Following this, cells receiving surface stains were FC blocked (CD16/32, 1:100 BD Biosciences), incubated with primary antibodies, and fixed with 1% formaldehyde, and resuspended in FACS Buffer. Samples were covered in aluminum foil and stored at 4°C until analysis.<br>For intracellular staining, if stimulation and golgi blocking were required, samples were incubated for 4 hours in complete RPMI at 37°C in a humidified incubator, with PMA (100ng/mL), ionomycin (1µg/mL), Brefeldin A (Biolegend), and Monensin (Biolegend). Following this, samples were surface stained as above before undergoing fixitation and permeabilization (eBioscience) in accordance with the manufacturers protocol. Following this samples were stained with intracellular antibodies, washed and resuspended in FACS Buffer. Samples were covered in aluminum foil and stored at 4°C until analysis (less than 24 hours later).
Instrument	Data were acquired on a Becton-Dickinson LSR II.
Software	FlowJo 10.6.1 software (FlowJo, LLC)
Cell population abundance	In some cases post-sort fraction were re-analyzed by flow-cytometry to confirm >=95% purity.
Gating strategy	All flow events were gated with FSC/SSC to remove the cell debris. Then, single cell events were gated with FSC-A/FSC-W and SSC-A/SSC-W.

☒ Tick this box to confirm that a figure exemplifying the gating strategy is provided in the Supplementary Information.
